# Twin arginine translocation, ammonia incorporation, and polyamine biosynthesis are crucial for *Proteus mirabilis* fitness during bloodstream infection

**DOI:** 10.1371/journal.ppat.1007653

**Published:** 2019-04-22

**Authors:** Chelsie E. Armbruster, Valerie S. Forsyth, Alexandra O. Johnson, Sara N. Smith, Ashley N. White, Aimee L. Brauer, Brian S. Learman, Lili Zhao, Weisheng Wu, Mark T. Anderson, Michael A. Bachman, Harry L. T. Mobley

**Affiliations:** 1 Department of Microbiology and Immunology; Jacobs School of Medicine and Biomedical Sciences; State University of New York at Buffalo; Buffalo, NY, United States of America; 2 Department of Microbiology and Immunology; University of Michigan Medical School; Ann Arbor, MI, United States of America; 3 Department of Biostatistics; University of Michigan School of Public Health; Ann Arbor, MI, United States of America; 4 Department of Computational Medicine & Bioinformatics; University of Michigan Medical School; Ann Arbor, MI, United States of America; 5 Department of Pathology; University of Michigan Medical School; Ann Arbor, MI, United States of America; University of California Davis School of Medicine, UNITED STATES

## Abstract

The Gram-negative bacterium *Proteus mirabilis* is a common cause of catheter-associated urinary tract infections (CAUTI), which can progress to secondary bacteremia. While numerous studies have investigated experimental infection with *P*. *mirabilis* in the urinary tract, little is known about pathogenesis in the bloodstream. This study identifies the genes that are important for survival in the bloodstream using a whole-genome transposon insertion-site sequencing (Tn-Seq) approach. A library of 50,000 transposon mutants was utilized to assess the relative contribution of each non-essential gene in the *P*. *mirabilis* HI4320 genome to fitness in the livers and spleens of mice at 24 hours following tail vein inoculation compared to growth in RPMI, heat-inactivated (HI) naïve serum, and HI acute phase serum. 138 genes were identified as *ex vivo* fitness factors in serum, which were primarily involved in amino acid transport and metabolism, and 143 genes were identified as infection-specific *in vivo* fitness factors for both spleen and liver colonization. Infection-specific fitness factors included genes involved in twin arginine translocation, ammonia incorporation, and polyamine biosynthesis. Mutants in sixteen genes were constructed to validate both the *ex vivo* and *in vivo* results of the transposon screen, and 12/16 (75%) exhibited the predicted phenotype. Our studies indicate a role for the twin arginine translocation (*tatAC*) system in motility, translocation of potential virulence factors, and fitness within the bloodstream. We also demonstrate the interplay between two nitrogen assimilation pathways in the bloodstream, providing evidence that the GS-GOGAT system may be preferentially utilized. Furthermore, we show that a dual-function arginine decarboxylase (*speA*) is important for fitness within the bloodstream due to its role in putrescine biosynthesis rather than its contribution to maintenance of membrane potential. This study therefore provides insight into pathways needed for fitness within the bloodstream, which may guide strategies to reduce bacteremia-associated mortality.

## Author summary

*Proteus mirabilis* is a common causative agent of catheter-associated urinary tract infections (CAUTI), one of the most prevalent healthcare-associated infections. Multidrug-resistant isolates of *P*. *mirabilis* are increasingly common and pose a severe challenge for treatment of CAUTI and secondary bloodstream infections, a common complication of CAUTI. However, there is a knowledge gap regarding the pathogenesis of *P*. *mirabilis* during bacteremia. We utilized a library of transposon mutants coupled with next-generation sequencing to perform a genome-wide assessment of the fitness requirements of *P*. *mirabilis* during incubation in serum *ex vivo* compared to during experimental bacteremia. This approach led to identification of a cohort of genes that are specifically important for establishing infection in both the liver and the spleen *in vivo*, several of which have also been implicated in secondary bacteremia following experimental CAUTI. Further exploration of genes critical for bloodstream pathogenesis could give rise to targets for novel antimicrobial therapies and potentially vaccines.

## Introduction

The Gram-negative bacterium *Proteus mirabilis* commonly causes catheter-associated urinary tract infection (CAUTI), particularly in the elderly and in healthcare facilities [1–5]. Consequences of *P*. *mirabilis* CAUTI can include infection of the kidneys, urinary stone formation due to bacterial urease (urolithiasis), permanent renal damage, dissemination of bacteria to the bloodstream (bacteremia and/or sepsis), and possibly death [5–9]. In healthcare facilities including nursing homes, CAUTI is the most common cause of secondary bacteremia, which is associated with a one-year mortality rate of 10–13% in most settings, but can be as high as 66% [6, 7, 10, 11].

Prior studies have shown that *P*. *mirabilis* is the causative agent in 13–21% of bacteremias experienced by nursing home residents, and the urinary tract is the predominant source of these CAUTI-associated bacteremias [12–18]. There are also increasing reports of antibiotic-resistant *P*. *mirabilis* isolates, including production of extended-spectrum β-lactamases (ESBLs) and carbapenemases [19–22], which is problematic as the mortality rate for ESBL-positive *P*. *mirabilis* bloodstream infections is significantly higher than that of ESBL-negative *P*. *mirabilis* isolates [23–25]. In North America, the percent of imipenem-resistant *P*. *mirabilis* bacteremia isolates increased from 0.2% to 35.3% between 2008 and 2012 [26]. ESBL-positive *P*. *mirabilis* isolates also tend to be multidrug-resistant (MDR). For instance, approximately 40% of 405 *P*. *mirabilis* clinical urine isolates in Japan were ESBL producers, and roughly 70% of these isolates exhibited concomitant resistance to fluoroquinolones [20]. A recent study of 99 patients with *P*. *mirabilis* bloodstream infections highlighted the consequences of MDR infections, as the 21-day mortality rate for individuals with MDR *P*. *mirabilis* was 50%, compared to 19% of those with non-MDR strains [27]. Since there are no licensed vaccines against *P*. *mirabilis*, identification of novel, non-antibiotic targets of treatment would be advantageous.

Many studies have investigated potential virulence factors encoded by *P*. *mirabilis* and their contribution to pathogenicity in animal models of UTI and CAUTI [summarized in [5]], but there have been no direct experimental evaluations of *P*. *mirabilis* virulence factors for bloodstream infection. However, some UTI and CAUTI fitness factors also contribute to spleen colonization following infection of the urinary tract, which is indicative of secondary bacteremia. For instance, factors pertaining to defense against antimicrobial peptides are important for fitness in a mouse model of CAUTI, and disruption of the polymyxin resistance gene *arnA* results in a fitness defect in the urinary tract as well as in the spleen [28]. Nitrogen assimilation pathways also contribute significantly to experimental UTI and CAUTI, with the potential to impact bacteremia. Ammonia is the preferred nitrogen source of *P*. *mirabilis*, which encodes two ammonia incorporation systems: glutamine synthetase (*glnA*) and glutamate dehydrogenase (*gdhA*). Glutamine synthetase (*glnA*) is a critical fitness factor for urinary tract colonization in the CAUTI model and spleen colonization in mice that progressed to bacteremia [28], and glutamate dehydrogenase (*gdhA*) was previously shown to be important for colonization of the urinary tract and spleen in an ascending model of UTI [29].

We previously generated a genome-saturating library of transposon mutants in *P*. *mirabilis* CAUTI isolate HI4320 and utilized transposon insertion-site sequencing (Tn-Seq) to identify 629 genes encoding candidate fitness factors for colonization and survival in the catheterized bladder and/or kidneys of infected mice [28]. Many of these genes have the potential to be important for dissemination to the bloodstream from the urinary tract or for survival within the bloodstream. In this study, we applied Tn-Seq to directly identify *P*. *mirabilis* fitness factors that contribute to survival within the bloodstream *versus* those that are important for survival in serum *ex vivo*.

## Results

### Estimation of bottleneck and optimal transposon mutant pools

We previously determined that 34,249 transposon mutants are required for 99.99% probability of full genome coverage, based on the *P*. *mirabilis* HI4320 genome size [28, 30]. The optimal infectious dose of *P*. *mirabilis* HI4320 (wild type, or WT) was determined to be 1x10^7^ CFU/ml via intravenous inoculation to achieve spleen and liver colonization in 100% of inoculated mice with no mortality (S1A Fig). This dose results in lesions in some of the mice by 24 hours post-inoculation (hpi) and would likely be lethal if allowed to progress, although the majority of mice show no outward signs of disease. To assess potential bottlenecks in the mouse model of bacteremia that could result in decreased recovery of mutants due to factors unrelated to fitness, we performed a competition infection with a *P*. *mirabilis* mutant that has a neutral fitness phenotype compared to WT during bloodstream infection.

As blood plasma contains only ~3 mM urea [(100- to 1,000-fold less than in urine [31, 32]], we hypothesized that urease would not provide a significant advantage to *P*. *mirabilis* during direct bloodstream infection and that a urease mutant would exhibit a neutral phenotype ideal for bottleneck assessment. We therefore compared a *P*. *mirabilis* urease mutant (*ureF*) to the WT strain in the bacteremia infection model (S1B Fig). The mutant achieved a similar bacterial burden as WT in all organs, indicating that it would be suitable for bottleneck assessment. Mice were next inoculated with the *ureF* mutant and WT *P*. *mirabilis* in ratios of 1:1, 1:1000, and 1:10,000 for bottleneck estimation (S1C and S1D Fig). The median colonization density for all mice was ~2x10^7^ CFU/gram of tissue in the liver, 1x10^6^ CFU/g spleen, and 1x10^3^ CFU/g kidneys, indicating that a sufficient colonization density for Tn-Seq is achieved in the spleen and liver but not the kidneys (S1C Fig). For bottleneck assessment, a competitive index was calculated based on the ratio of *ureF*:WT from the liver and spleen compared to the input ratio. While there was more variability in the competitive index for mice inoculated with a 1:10,000 ratio of *ureF*:WT than the 1:1 or 1:1,000 ratios, the competitive index was not found to be significant for any ratio, indicating that there was not a significant bottleneck (S1D Fig). Based on these data, the use of 5x10^4^ transposon mutants would be suitable for Tn-Seq in the bacteremia model, providing 50x coverage of each mutant for an inoculum of 1x10^7^ CFU. A transposon mutant pool was therefore generated by combining the 5 pools of 10,000 mutants each that we previously validated and utilized in the CAUTI Tn-Seq study [28].

### Identification of infection-specific fitness factors

A schematic of the Tn-Seq experimental setup is provided in S2 Fig. Ten mice were inoculated intravenously with the *P*. *mirabilis* pool of transposon mutants to assess fitness factors for survival within the bloodstream, as measured by recovery from spleens and livers 24 hours post-inoculation. All 10 mice exhibited adequate spleen and liver colonization for analysis (S3 Fig). Concurrently, the transposon mutant pool was also subjected to three *in vitro* conditions to facilitate identification of fitness factors that are infection-specific, and therefore have defects in the bacteremia screen but are not required for fitness during incubation in serum *ex vivo*. The *in vitro* conditions included for this assessment were as follows: 1) RPMI medium alone (labeled “RPMI”), 2) RPMI medium with 50% heat-inactivated naïve mouse serum (generated from CBA/J mice, labeled “Naïve”, and 3) RPMI with 50% heat-inactivated acute-phase serum (generated from CBA/J mice 5 hours after intraperitoneal injection with heat-killed *P*. *mirabilis*, labeled “APS”). Heat-inactivated serum was utilized for these studies to remove heat-labile antimicrobial compounds, allowing genes involved in tolerance of these factors to be retained as potentially infection-specific.

**Fig 1:**
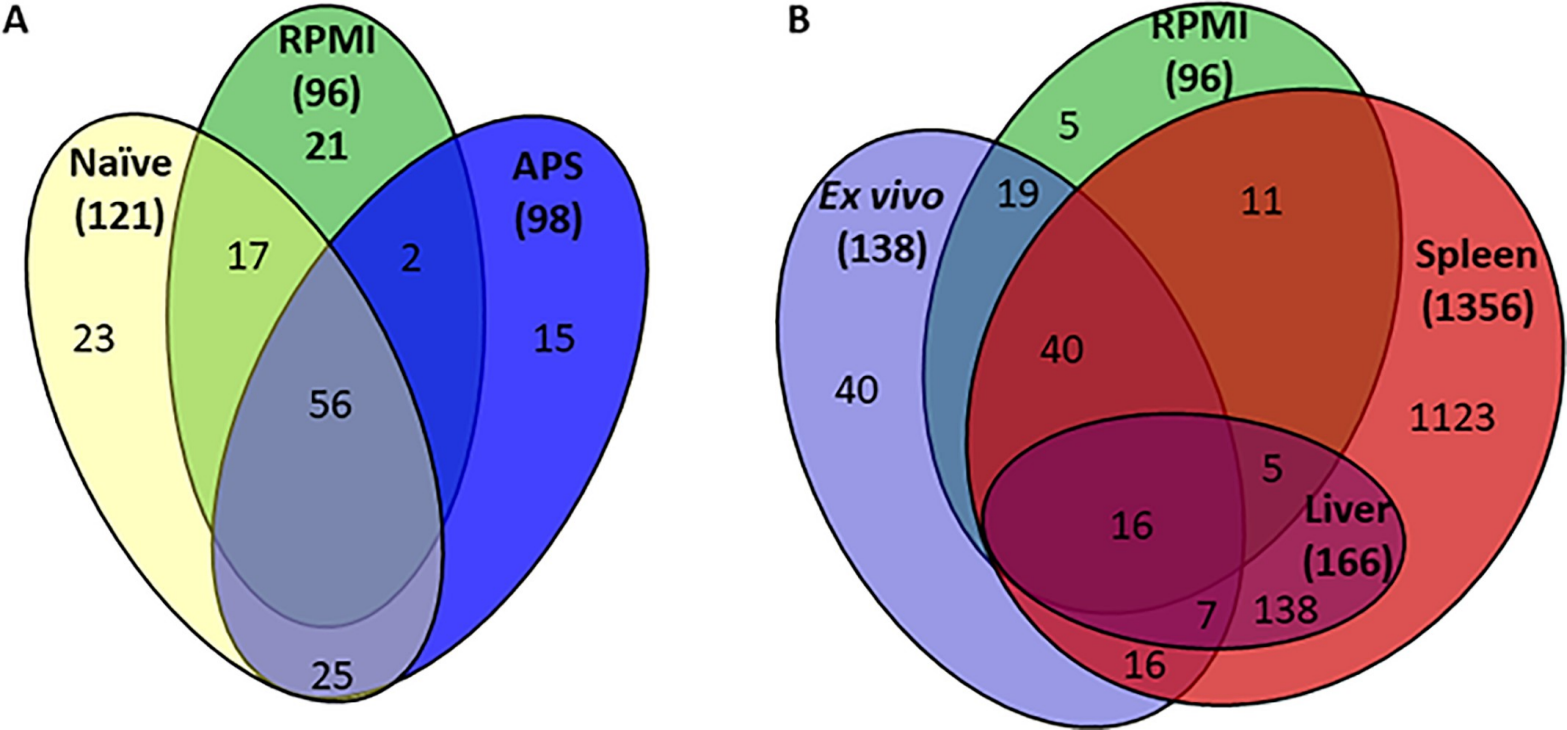
Distribution of Tn-Seq candidate fitness factors in vitro compared to in vivo. (A) A Venn diagram illustrating the number of overlapping candidate *P. mirabilis* fitness factors among three growth conditions: 1) RPMI medium alone (RPMI), 2) RPMI medium with 50% heat-inactivated naïve mouse serum (Naïve), and 3) RPMI with 50% heat-inactivated acute-phase serum (APS). (B) A Venn diagram depicts the number of overlapping candidate *P. mirabilis* fitness factors among four growth conditions: 1) RPMI medium with either 50% heat-inactivated naïve mouse serum or 50% heat-inactivated acute-phase serum (Ex vivo), 2) RPMI medium alone, 3) mouse spleen, and 4) mouse liver. Numbers in parentheses represent the total number of candidate fitness factors for each group.

**Fig 2:**
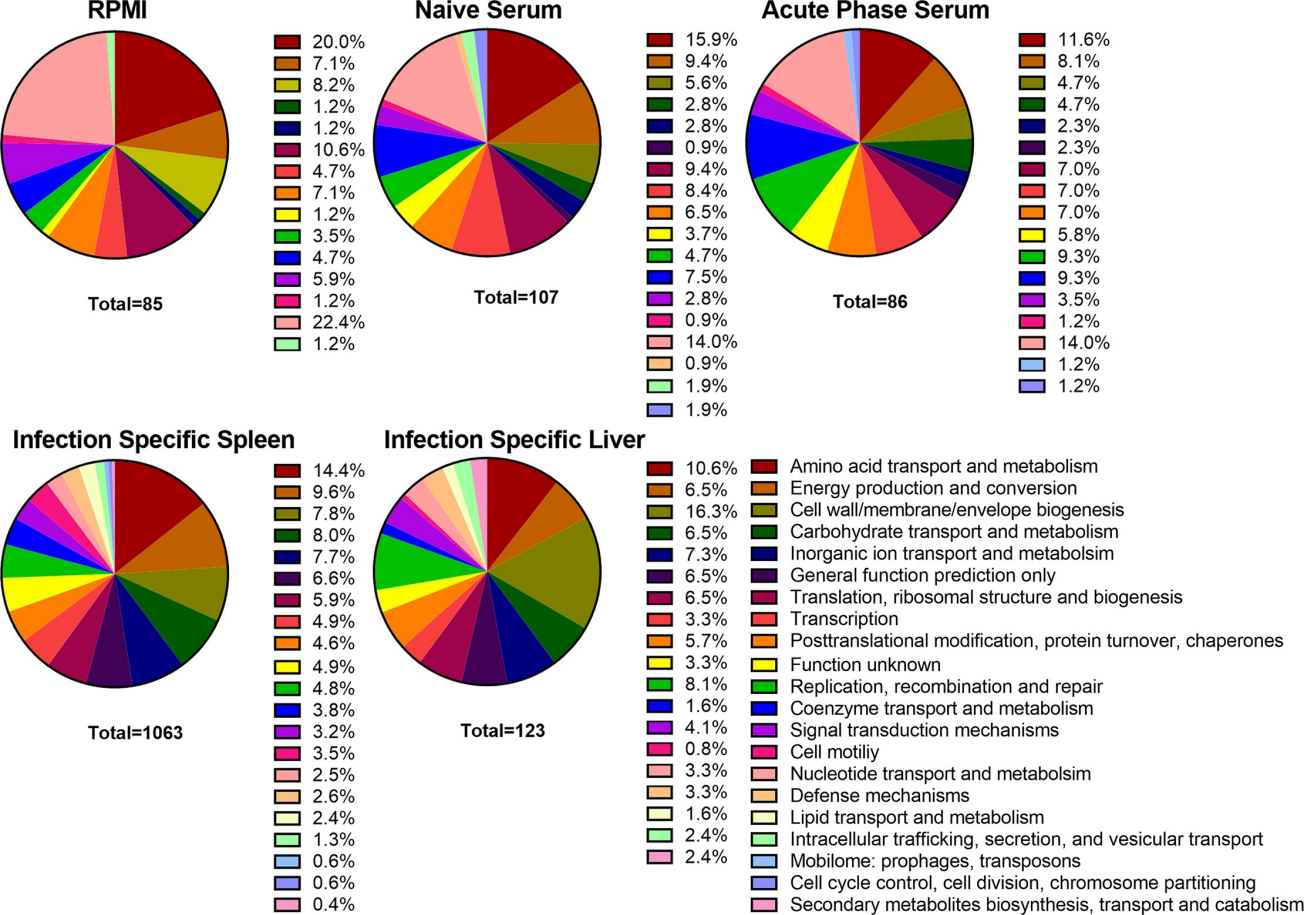
COG categories of Tn-Seq candidate fitness factors. The percentage of candidate *P. mirabilis* fitness factors required for growth in RPMI, 50% heat-inactivated naive mouse serum and 50% heat-inactivated acute phase mouse serum belonging to each Cluster of Orthologous Groups of proteins (COG). The percentage of candidate infection-specific *P. mirabilis* fitness factors during colonization of the murine spleen and liver 24 hours post-inoculation belonging to each COG, for which only genes identified during infection that had no defect in vitro are represented. The total number of fitness factors for each group with an identified COG category is listed under its respective pie chart.

Each gene was assigned a fitness index for each condition based on the number of unique insertion-sites within that gene and the depth of reads at each site for a given condition relative to the input samples. Genes were considered to be candidate fitness factors based on having an adjusted *P-*value <0.05, and a ratio of input/output ≥2-fold. Fitness factors for spleen or liver colonization that were not identified as important for growth in either serum condition *in vitro* were considered to be infection-specific. The full dataset is provided in S1 Table and an overview of the fitness factors identified for each condition is provided in Figs 1 and 2, which will be discussed in detail below.

A total of 96 genes (2.5% of the 3747 genes encoded in the *P*. *mirabilis* HI4320 genome) were fitness factors for growth in RPMI in the absence of mouse serum (S2 Table, and summarized in Fig 1A and 1B). RPMI fitness factors were most commonly associated with nucleotide transport and metabolism (22.4%), amino acid transport and metabolism (20.0%), translation, ribosomal structure and biogenesis (10.6%), and cell wall/membrane/envelope biogenesis (8.2%) (Fig 2). 75 of these 96 genes (78%) were also fitness factors in mouse serum (either naïve or APS, shown in S3 Table and Fig 1A and 1B), indicating that they are likely required for growth in RPMI and the presence of serum cannot complement growth. These factors primarily pertained to amino acid transport/metabolism and nucleotide transport/metabolism, and include: glutamine synthetase (*glnA*), glutamate 5-kinase and glutamate 5-semialdehyde dehydrogenase (*proAB*), aspartate-ammonia ligase (*asnA*), pyruvate dehydrogenase (*aceEF*), purine metabolic genes (*guaAB*, *purC*, *purNM*, *purF*, *purK*, *purH*, *purD*, and *purA*), and pyrimidine metabolic genes (*pyrF* and *pyrC*). This group also contains a stringent starvation protein (*sspA*), carbon storage regulator (*csrA*), and RNA polymerase σ^54^ (*rpoN*). 21 genes (21%) were identified as only being important for survival in RPMI but not in the presence of either naïve or acute-phase serum, indicating that components of mouse serum can complement the defects of these mutants in RPMI. Included in this list were genes involved amino acid transport and metabolism (*carAB*, *thrC*, *usg*, and *speB*), transport of magnesium (PMI1580) and vitamin B12 (*btuC*), and factors involved in cell wall synthesis (*rfaA*, *rfaD*, *rfaF*, and *wabG*). Interestingly, there were no fitness factors related to defense mechanisms or lipid transport in the RPMI condition without mouse serum, indicating that these factors are only important in the presence of host components.

138 genes (3.7%) were identified as *ex vivo* fitness factors in 50% mouse serum (S3 Table and Fig 1A and 1B). 121 genes were fitness factors in naïve mouse serum, of which 107 could be assessed based on their cluster of orthologous group function (COG). These genes were primary involved in amino acid transport and metabolism (15.9%), nucleotide transport and metabolism (14.0%), energy production and conversion (9.4%), and translation, ribosomal structure and biogenesis (9.4%), indicative of an increased need for transcriptional and translational machinery during growth in serum as compared to the medium in which the transposon library was generated (Fig 2). 73/121 (60.3%) were also important for growth in RPMI alone, while 48 (39.7%) were specific fitness factors for growth in serum. Of these 48 serum-specific fitness factors, 23 (47.9%) were only identified as fitness factors in naïve serum, while the remaining 25 were also identified in acute-phase serum.

For acute-phase serum (APS), 98 genes were identified as fitness factors (S3 Table and Fig 1A and 1B). 86 of these genes could be assessed based on their COG function, and the most highly represented categories include: nucleotide transport and metabolism (14%), amino acid transport and metabolism (11.6%), replication, recombination and repair (9.3%), and coenzyme transport and metabolism (9.3%, Fig 2). Compared to the naïve serum fitness factors, there was a greater proportion of genes involved in replication, recombination, and DNA repair, coenzyme transport, and coenzyme metabolism in the APS. 58 of the 98 genes for fitness in APS (59%) were also important for growth in RPMI alone, while 40 were serum-specific. Of the 40 serum-specific factors, 25 (62.5%) were also identified in naïve serum.

By excluding the fitness factors important for growth in RPMI, a total of 63 genes were identified as *in vitro* serum-specific fitness factors (S4 Table and Fig 1A). This group includes members of the chorismate/shikimate pathway (*aroB*, *aroC*, *aroE*, and *aroK*) for synthesis of aromatic amino acids and siderophores, hemin receptors *hmuR1* and *hmuR2*, the *exbBD* outer membrane transport proteins along with *tonB* and *tolC*, the Rnf electron transport complex (*rnfADEG*), and numerous metabolic enzymes (triosephosphate isomerase [*tpiA*], glucose-6-phosphate isomerase [*pgi*], 6-phosphofructokinase [*pfkA*], 6-phosphogluconolactonase [pgl], and the leucine-responsive transcriptional regulator *lrp*). This category also includes colicin (*cvpA*), and two components of the “high frequency of lysogenization” locus (*hflCK*), which has been linked to tolerance of cell membrane and cell envelope stress in other bacterial species [33]. There are also several factors involved in replication, recombination, and repair, such as *recA* recombinase, DNA polymerase III subunits *holD* and *holC*, tyrosine recombinase *xerD*, and a putative ATP-dependent DNA helicase (*dinG*).

With the *in vitro* fitness factors characterized, we next analyzed the spleen and liver samples to identify infection-specific fitness factors for experimental bacteremia. In total, 1,356 genes (36% of the genome) were identified as candidate fitness factors from spleen samples, 166 of which were also candidate fitness factors from liver samples (Fig 1B). By subtracting out the candidate fitness factors that also exhibited defects in mouse serum *ex vivo*, there were 1,278 candidate infection-specific fitness factors, 1,135 of which were specific to spleen samples and 143 were fitness factors for both spleen and liver colonization (S5 Table). There were no liver-specific fitness factors (as illustrated in Fig 1B), indicating that both organs harbor bacteria that have likely undergone the same selection process. In addition to identifying genes with potential fitness defects, Tn-Seq can also reveal if loss of a gene provides a fitness advantage to a bacterium. There were 106 genes that had a fitness advantage in the liver of ≥2-fold compared to the input, but only 2 were statistically significant: a tRNA (PMIt055) and a plasmid gene PMIP18. In the spleen, 23 genes had a fitness advantage of ≥2-fold compared to the input, but none were statistically significant. Thus, our analysis revealed only 2 genes for which loss may provide *P*. *mirabilis* with a fitness advantage during bacteremia.

Regarding the infection-specific fitness factors for colonization of both spleen and liver, 71/143 genes were predicted to be contained within operons. We therefore assessed the other genes in each operon for fitness defects in any of the conditions tested (S5 Table). On average, 44% of the genes in each represented operon (range 7–100%) were identified as infection-specific fitness factors for both the liver and spleen. This value increases to 78% (range 33–100%) for infection-specific fitness factors for the spleen only. Furthermore, only 2/71 operons (3%) contained genes identified as having defects in serum or RPMI *in vitro*. Thus, this approach appears to accurately identify gene sets that contribute to *P*. *mirabilis* fitness during infection but not during incubation in serum *in vitro*.

Of the 143 infection-specific fitness factors for both spleen and liver, 117 (82%) were previously identified as potentially contributing to infection within the urinary tract: 116 were identified as fitness factors during CAUTI infection by Tn-Seq [28], 29 were identified as being upregulated during ascending UTI [29], and 8 were identified as fitness factors during ascending UTI [5]. Spleen-specific fitness factors primarily pertained to amino acid transport and metabolism (14.4%), energy production and conversion (9.6%), and carbohydrate transport and metabolism (8.0%) functional categories (Fig 2). Infection-specific fitness factors common to colonization of the spleen and liver pertained to cell wall/membrane/envelope biogenesis (16.3%), amino acid transport and metabolism (10.6%), inorganic ion transport and metabolism (7.3%), and replication, recombination and repair (8.1%) (Fig 2). The fold-change in abundance of mutants representing infection-specific fitness factors in both the spleen and liver ranged from 88.8 to 4.2, with 5 of the top 10 fitness factors within the cell wall/membrane/envelope biogenesis category.

It is not surprising that survival during bloodstream infection is highly dependent on the integrity of the cell wall, as contact with innate immune defenses is imminent in the bloodstream environment. Examples of infection-specific genes involved in cell wall biogenesis are *arnABC*, which modifies the charge of LPS and is important for resistance against polymyxin and cationic peptides, as well as the adjacent gene encoded by PMI_RS05085 (PMI1046) that deformylates a component of lipid A. Six members of the *waa* gene cluster involved in synthesis of the LPS core were also identified as infection specific factors, including three glycosyl transferases (PMI_RS15630/PMI3163, PMI_RS15635/PMI3163, PMI_RS15620/PMI3159). All of these genes were previously implicated as being *in vivo* fitness factors during both ascending UTI and CAUTI [28, 34]. In addition, five genes proposed to be involved in capsule biosynthesis were identified as infection specific fitness factors in the spleen (PMI_RS15765/PMI3188, PMI_RS15785/PMI3192, PMI_RS15800-PMI_RS15810/PMI3195-97). The fitness factor with the greatest fold-change in abundance was *ompF*, an outer membrane porin previously identified as being a fitness factor during CAUTI [28]. PMI_RS16875/PMI3390, a putative autotransporter about which little is known, was another infection-specific fitness factor that was previously identified as being differentially expressed during ascending UTI as well as a fitness factor for both ascending UTI and CAUTI [28, 29, 34].

**Fig 3:**
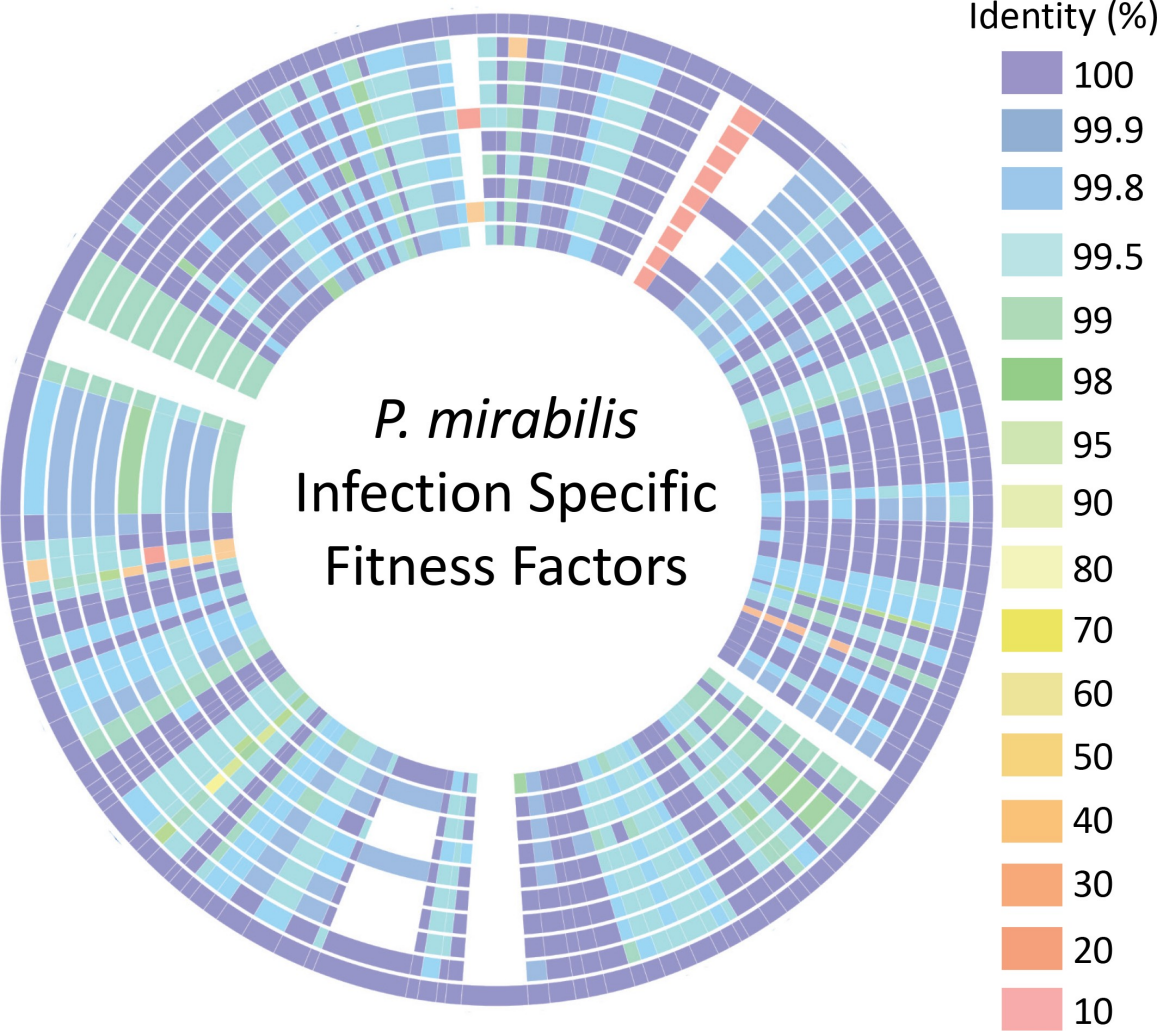
Conservation of infection-specific fitness factors between *P. mirabilis* isolates. The amino acid sequences available on PATRIC of 143 infection-specific fitness factors identified in *P. mirabilis* HI4320 were arranged end-to-end on the outer ring and compared to other *P. mirabilis* proteomes to identify homologs in each of these strains. The level of amino acid sequence identity is indicated by color. Tracks from outside to inside represent the following *P. mirabilis* isolates: 1) HI4320; 2) AR_0159; 3) PM_125; 4) PM_178; 5) MH13-009N; 6) AOUC-001; 7) NLAE-zl-C285; 8) CYPM1; 9) 1330_PMIR; 10) 429_PMIR.

Notably, the infection-specific fitness factors exhibit a high degree of conservation between *P*. *mirabilis* strains. 129 of 143 (90%) infection-specific factors that were identified in strain HI4320 are more than 90% homologous to genes present in 9 other *P*. *mirabilis* strains with available complete genome sequences (Fig 3 and S6 Table). Only four genes were completely unique to strain HI4320: a hypothetical membrane protein (PMI_RS12130/PMI2454), a DNA methyl transferase (PMI_RS12255/PMI2479), a type II restriction endonuclease (PMI_RS15540/PMI3141) and a putative RNA helicase (PMI_RS12250/PMI2478). Another seven genes were present in other strains but with less than 90% homology, including RNA polymerase associated protein (*rapA*), lipopolysaccharide core heptosyltransferase III (PMI_RS15665/PMI3168), type VI secretion tip protein (*vgrG*), heptosyl LPS alpha 1,3-glucosyltransferase (*waaG*), vitamin B12 receptor (*btuB*) and two hypothetical proteins PMI_RS15640/PMI3163 and PMI_RS18555/PMIP09. Thus, there is a remarkable degree of conservation between strains for the genes identified as infection-specific fitness factors in *P*. *mirabilis* HI4320, which has also been observed in the closely related species *Citrobacter freundii*, with 82% of fitness factors having >90% similarity between isolates [35].

### Validation of candidate fitness factors

Six mutants were generated in genes identified as infection-specific fitness factors in both the spleen and liver for initial validation of the screen: a polymyxin resistance protein *(arnA*, 4/7 genes in this operon were infection-specific fitness factors), vitamin B12 transporter (*btuB*, not predicted to be part of an operon), propanediol utilization protein (*cutC*, not predicted to be part of an operon), glutamate synthase (*gltB*, 2/2 genes in this operon were infection-specific fitness factors), arginine decarboxylase (*speA*, not predicted to be part of an operon), and sec-independent protein translocation (*tatC*, 1/3 genes in this operon were infection-specific). We also generated mutants for three genes identified as fitness factors *in vitro* in mouse serum: aspartate-ammonia ligase (*asnA*, fitness defect in RPMI and serum), colicin (*cvpA*, fitness defect in serum), and a regulator of the FtsH protease (*hflK*, fitness defect in serum).

**Fig 4:**
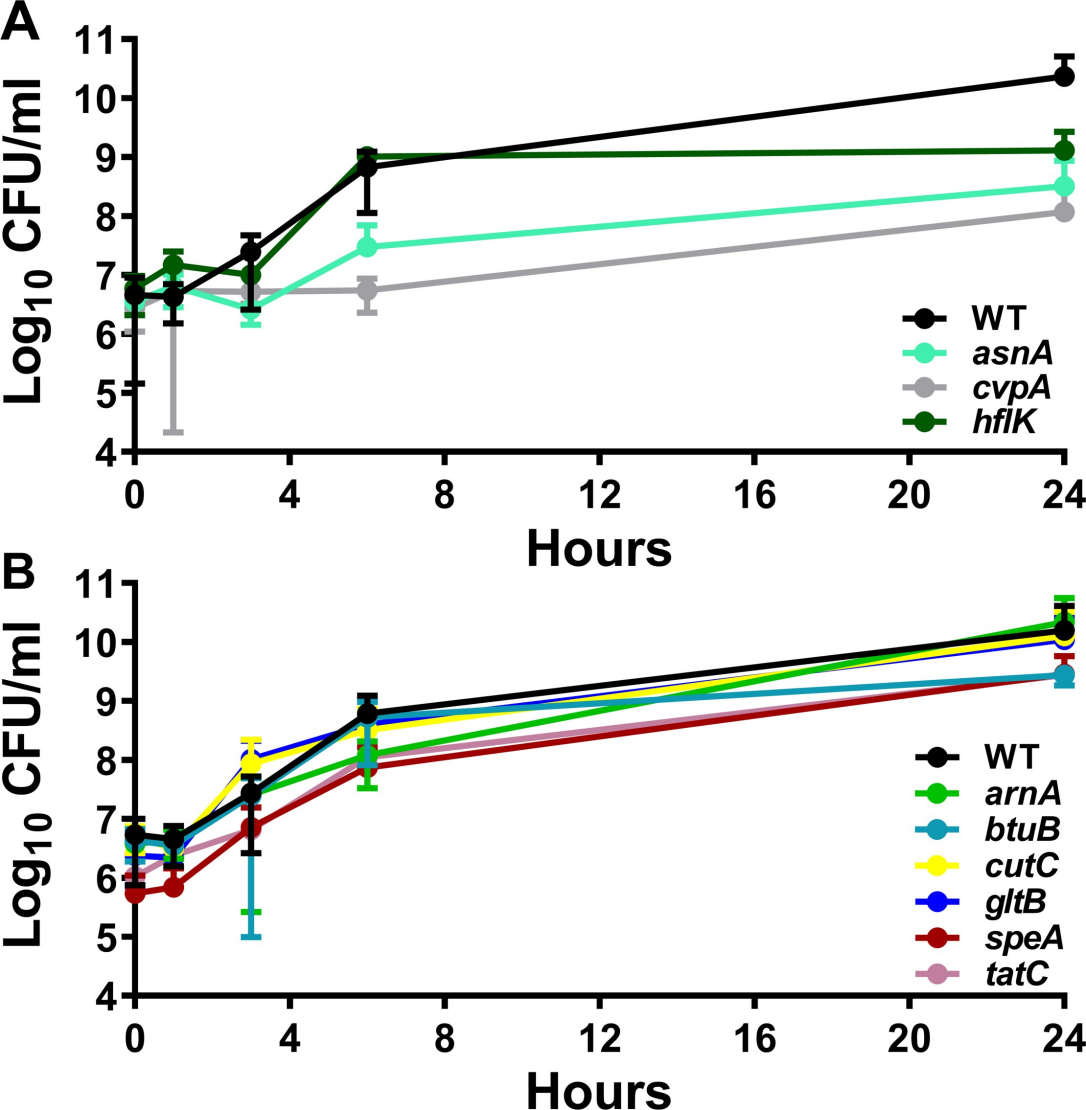
Serum growth characteristics of candidate fitness factor mutants. Growth of wild-type *P. mirabilis* and mutants over the course of 24 hours during static incubation at 37°C with 5% CO2 in 50% naive mouse serum for genes that are (A) predicted to have a defect in serum (*asnA, cvpA, hflK*) or (B) candidate infection specific fitness factors (*arnA, btuB, cutC, gltB, speA, tatC*). Cultures were sampled at 0, 1, 3, 6, and 24 hours for enumeration of CFUs on LB agar. Error bars represent the mean ± standard deviation from three biological replicates.

Survival in mouse serum *in vitro* was first assessed to determine if any of the mutants were susceptible to serum killing. These studies utilized naïve mouse serum that had not been heat-inactivated in order to retain heat-labile antimicrobial compounds as a more stringent assessment of survival. All three of the mutants predicted to have defects *in vitro* recapitulated the Tn-Seq results by exhibiting decreased CFUs compared to WT during the time course (*P*<0.001 by two-way ANOVA, Fig 4A), while none of the candidate infection-specific mutants exhibited growth defects in 50% naïve mouse serum (Fig 4B). Growth in LB medium was next assessed, and only the *asnA* mutant exhibited a slight defect (*P* = 0.0095 by two-way ANOVA, S4A and S4B Fig). In addition, growth was assessed in the minimal medium PMSM to uncover auxotrophy, and RPMI to recapitulate the Tn-Seq *in vitro* screen conditions. The candidate infection-specific factors were all able to reach the same level of saturation as WT in PMSM (S5D Fig) and RPMI (S4F Fig), although *btuB*, *gltB*, *speA* and *tatC* all had significant growth delays in RPMI (*P*<0.001 by two-way ANOVA). The *asnA* mutant was unable grow in PMSM unless supplemented with 10 mM asparagine (S4C Fig) and similarly exhibited a severe defect in RPMI (S4E Fig). Unexpectedly, the *cvpA* mutant was unable to grow in PMSM or RPMI, and the *hflK* mutant exhibited a defect in RPMI (*P*<0.007 by two-way ANOVA), which was not expected based on the Tn-Seq screen results. Taken together, 7/9 mutants (78%) recapitulated the expected *in vitro* phenotypes, 1 mutant (*hflK*) recapitulated the expected phenotypes in 3 out of 4 conditions, and 1 mutant (*cvpA*) recapitulated the expected phenotypes in 2 out of 4 conditions.

**Fig 5:**
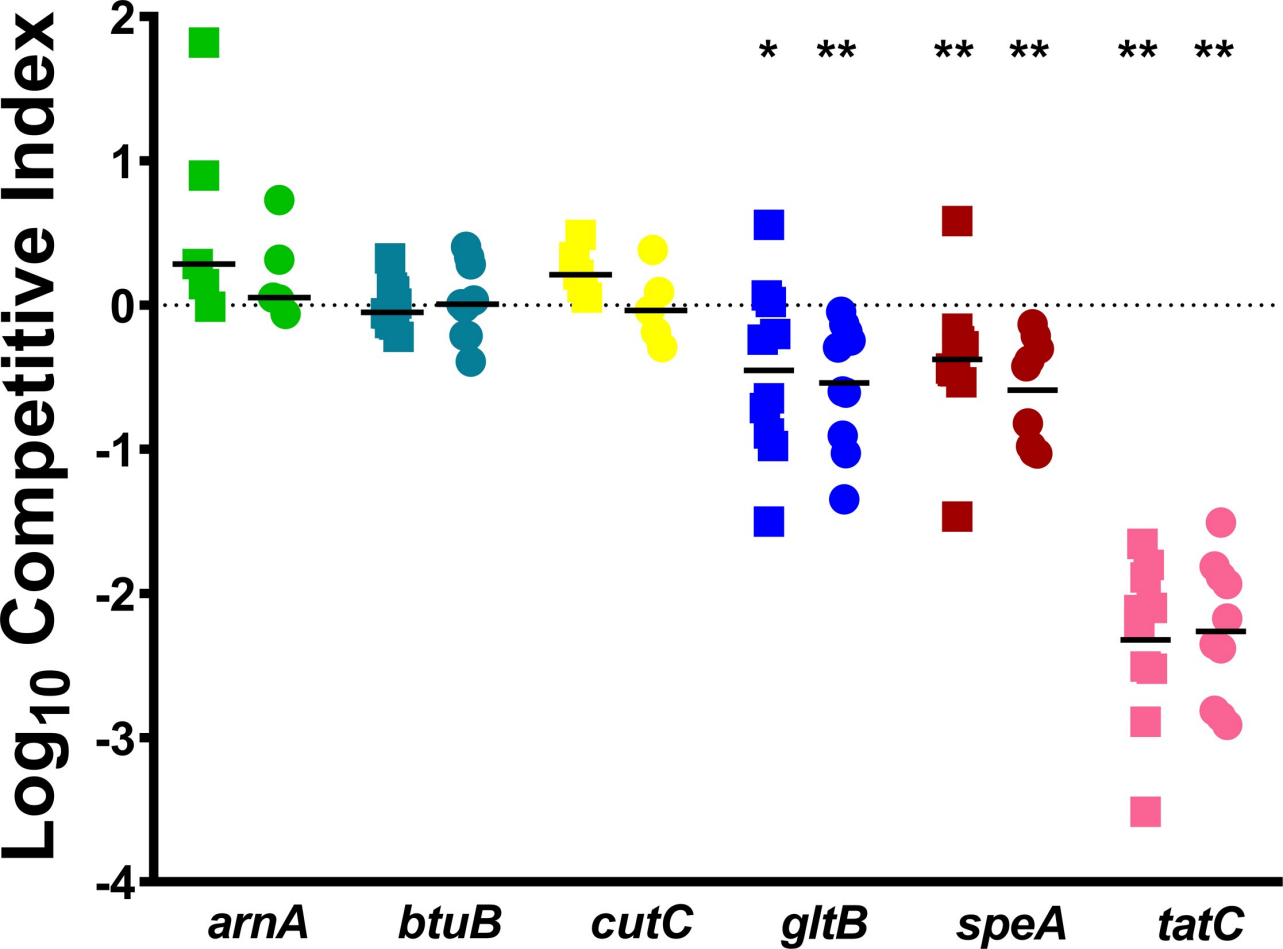
Disruption of *gltB, speA*, or *tatC* results in a fitness defect during direct co-challenge with WT *P. mirabilis*, while disruption of *arnA, btuB*, or *cutC* does not. CBA/J mice were inoculated via the tail vein with 1x10^7^ CFU of a 1:1 mixture of wild-type *P. mirabilis* and an isogenic mutant. Liver and spleen were harvested from mice 24 hours post-inoculation, homogenized, and plated on LB agar and LB agar with kanamycin. A competitive index (CI) was calculated for each mutant on a per-mouse basis for the liver (square) and spleen (circle) from the ratio of mutant to wild-type recovered from the organ divided by the ratio of mutant to wild-type in the inoculum (see Materials and Methods). Each data point represents the Log10 CI from an individual mouse. Solid lines represent the median. Dashed lines indicate a competitive index of 1, or a 1:1 ratio of mutant to wild-type. * P < 0.05 and ** P < 0.01 as determined by Wilcoxon signed rank test.

We next assessed the six candidate infection-specific fitness factors by direct co-challenge with WT *P*. *mirabilis* during bloodstream infection. Mice were inoculated with a 1:1 mixture of mutant:WT, the infection was allowed to progress for 24 hours, and CFUs of mutant and WT were determined for the liver and spleen of each mouse. The *gltB*, *speA*, and *tatC* mutants all recapitulated the Tn-Seq screen predictions by exhibiting fitness defects in the liver and the spleen (Fig 5). However, despite having severe defects in the Tn-Seq screen (16–41 fold-change in the spleen and 7–23 fold-change in the liver), the *arnA*, *btuB*, and *cutC* mutants did not appear to significantly contribute to fitness within the bloodstream during direct co-challenge as none of the mutants were significantly outcompeted by WT in any organ (Fig 5). This was particularly unexpected for *arnA*, because disruption of this gene was previously determined to result in a fitness defect in the spleen during CAUTI [28]. Taken together, 3/6 (50%) of the genes chosen for initial validation studies *in vivo* recapitulated the expected phenotypes.

This discrepancy could be due to a combination of factors, including the coverage of TA insertion sites within these genes, the specific gene location where a kanamycin resistance cassette was inserted during generation of TargeTron mutants *vs*. the transposon insertion sites present in the input pool for Tn-Seq, the fold-change and *P* value cutoffs used in the analysis, and the 1:1 ratio used for the co-challenge *vs*. the ~1:50,000 ratio present during the Tn-Seq screen. It is also notable that the genes that failed to validate were either not contained within an operon (*btuB* and *cutC*), or were within a fairly large operon where only half of the genes were predicted to have fitness defects (*arnA*). Thus, our initial results clearly highlight the critical importance of validating Tn-Seq results by direct co-challenge, and underscore the utility of exploring the contribution of complete gene operons or functional pathways to pathogenesis. Based on the *in vivo* defects observed in the *tatC*, *gltB* and *speA* mutants, we therefore chose to investigate the contribution of the twin-arginine translocation system, nitrogen assimilation, and polyamine biosynthesis to *P*. *mirabilis* fitness within the bloodstream in greater depth.

### Contribution of the twin-arginine translocation (Tat) system to fitness in the bloodstream

The twin-arginine translocation (Tat) system is utilized by numerous Gram-negative bacterial species to translocate periplasmic proteins and enzymes, particularly those involved in binding redox cofactors [36]. A prior Tn-Seq study in *Citrobacter freundii* identified the Tat system and putative substrates as important for fitness within the bloodstream [35]. *P*. *mirabilis* HI4320 encodes a Tat system (*tatA*, *tatB*, *tatC*) with spleen defects ranging from 20–58 fold and liver defects ranging from 11–27 fold. However, only *tatC* was determined to have a statistically-significant defect (S5 Table). This system was also identified as a candidate fitness factor for kidney colonization during experimental CAUTI [28], suggesting that it contributes to *P*. *mirabilis* fitness during multiple infection types.

The Tat-substrate prediction software TatP [37] was used to identify putative Tat substrates encoded by *P*. *mirabilis* HI4320 to determine if multiple putative substrates are also likely infection-specific fitness factors. 485 possible Tat substrates were predicted based on potential cleavage sites, 20 of which had clear Tat motifs (S7 Table). Among these 20 candidate Tat substrates, 13 (65%) were candidate infection-specific fitness factors for bacteremia, and one (*sufI*) is a homolog of a predicted Tat substrate that contributed to *C*. *freundii* fitness during bacteremia [35]. We therefore sought to further explore the contribution of the Tat system to *P*. *mirabilis* fitness in the bloodstream using mutants in *tatC* and *tatA*.

**Fig 6:**
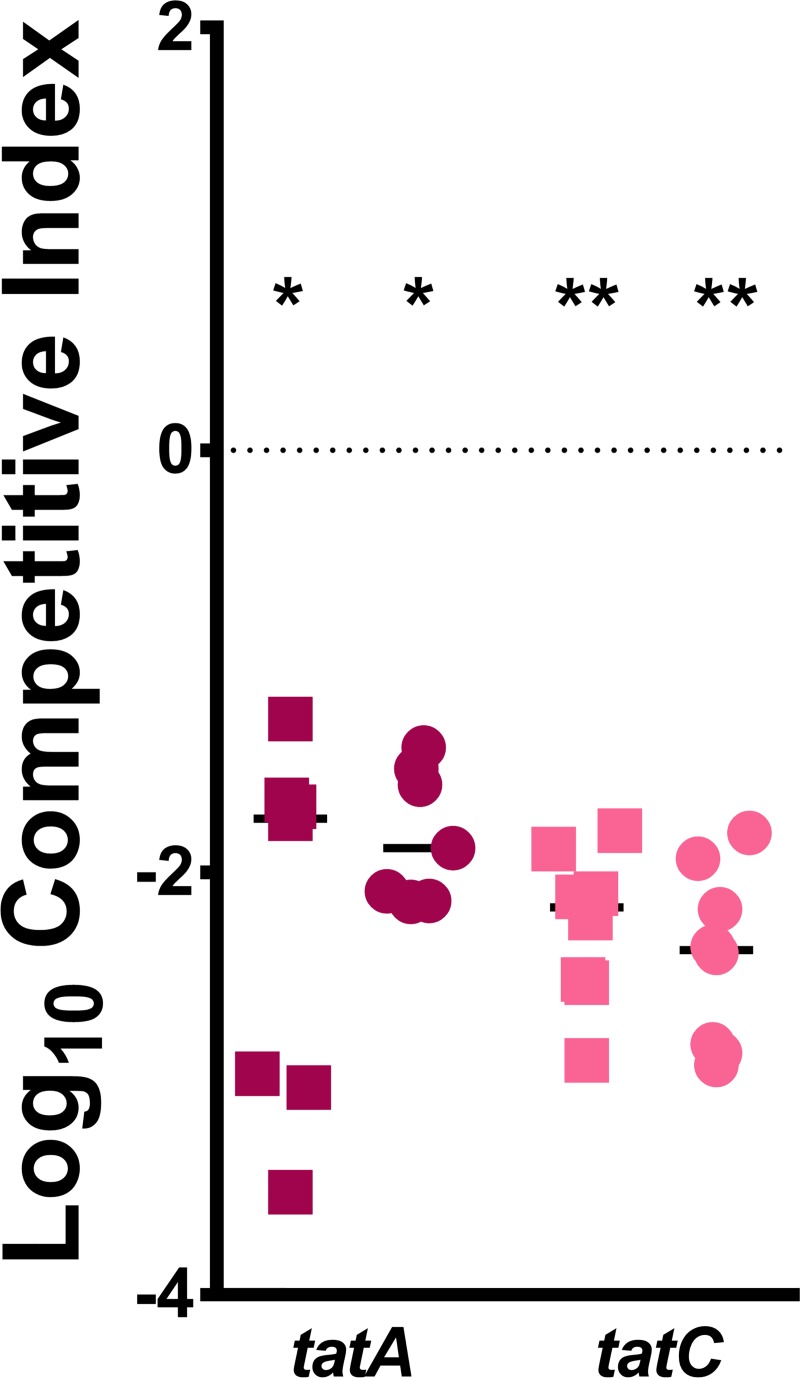
The Tat system contributes to *P. mirabilis* fitness during bacteremia. CBA/J mice were inoculated via the tail vein with 1x10^7^ CFU of a 1:1 mixture of wild-type *P. mirabilis* and an isogenic mutant. Liver and spleen were harvested from mice 24 hours post-inoculation, homogenized, and plated on LB agar and LB agar with kanamycin. A competitive index (CI) was calculated for each mutant on a per-mouse basis for the liver (square) and spleen (circle) as described above. Data for the *tatC* mutant are provided from Fig 5 for comparison. Each data point represents the Log10 CI from an individual mouse. Solid lines represent the median. Dashed lines indicate a competitive index of 1, or a 1:1 ratio of mutant to wild-type. * P < 0.05 and ** P < 0.01 as determined by Wilcoxon signed rank test.

The *tatA* mutant was first subjected to the same validation experiments as *tatC* to determine if both mutants exhibited comparable phenotypes. The *tatA* mutant had similar growth characteristics to the *tatC* mutant *in vitro*, including a pronounced lag phase in LB, PMSM, and RPMI (*P*<0.05 by two-way ANOVA, S5 Fig), and neither mutant exhibited a defect when incubated in naïve mouse serum. During co-challenge *vs*. WT *P*. *mirabilis* in the murine bacteremia model, both *tatA* and *tatC* were highly outcompeted by WT in liver and spleen, but the defect for the *tatC* mutant was more consistent and pronounced than *tatA* (Fig 6). This finding is in agreement with the results of the screen, and is consistent with the critical role of TatC in directly interacting with Tat signal peptides.

**Fig 7:**
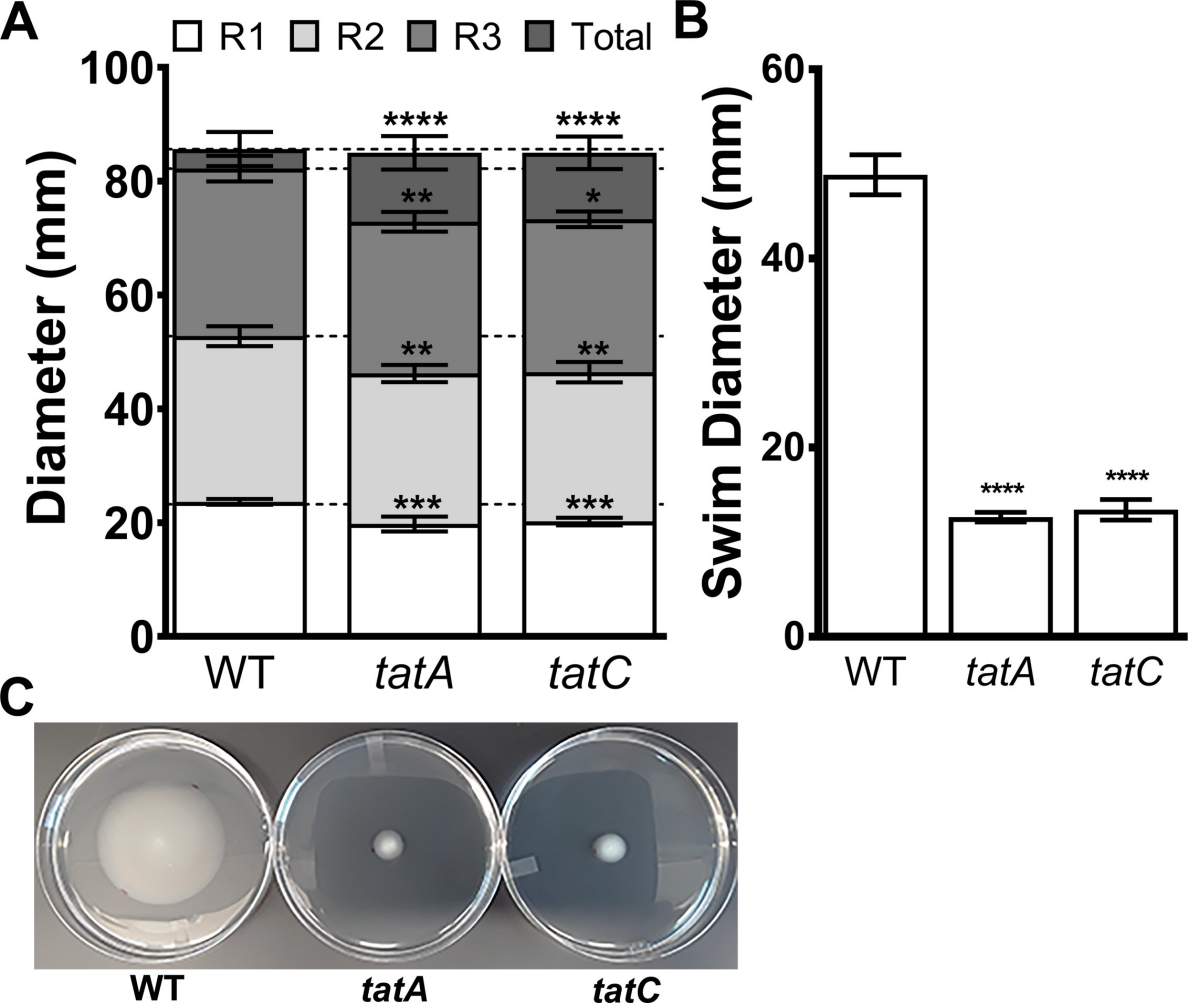
Disruption of the Tat system impacts swimming but not swarming motility. (A) Swarm diameters of the first (R1), second (R2), and third (R3) swarm rings and total swarm diameter of wild-type *P. mirabilis* HI4320 and isogenic mutants *tatA* and *tatC* inoculated on LB agar with 5g/L NaCl after 18 hours of incubation at 37°C. Dashed lines indicate average swarm ring diameter for WT. Error bars represent the mean ± standard deviation from 3 independent experiments with 3 replicates each. ** P < 0.01, *** P < 0.001, and ****P<0.0001 as determined by two-way ANOVA. (B) Swimming motility on MOT agar stab inoculated with wild-type *P. mirabilis* HI4320 and isogenic mutants *tatA* and *tatC*, incubated at 30°C for 18 hours. Error bars represent the mean ± standard deviation from 3 independent experiments with 4 replicates each. ****P<0.001 compared to WT as determined by Student’s t-test. (C) Representative image of wild-type *P. mirabilis* HI4320 and isogenic mutants *tatA* and *tatC* swimming on MOT agar.

*P*. *mirabilis* is well-known for its robust swimming and swarming motility, and mutations that disrupt the Tat system have been shown to perturb motility in *Escherichia coli*, *Yersinia pseudotuberculosis*, and *C*. *freundii* [35, 38, 39]. We therefore investigated the contribution of *tatA* and *tatC* to *P*. *mirabilis* motility (Fig 7). Both of the Tat mutants were capable of swarming to a similar extent as WT, but exhibited a minor decrease in the diameter of each swarm ring (Fig 7A). The Tat mutants also both exhibited a dramatic decrease in swimming motility in soft agar (Fig 7B and 7C). Thus, disruption of the Tat system abrogates *P*. *mirabilis* swimming motility, but not swarming motility, indicating a potential impact on factors important for chemotaxis rather than synthesis of flagella.

Notably, of the 56 genes in the flagella locus of *P*. *mirabilis* HI4320 [30], only 8 (14%) were candidate fitness factors in the spleen during bacteremia, not in the liver. Furthermore, none of the 20 putative Tat substrates with clear Tat motifs were directly related to flagellar biosynthesis, motility, or chemotaxis. It is therefore unlikely that motility contributes substantially to fitness within the bloodstream, and the defects observed for the Tat mutants most likely stem from loss of other secreted substrates, particularly those involved in metabolism. To confirm this hypothesis, we assessed the contribution of flagella to *P*. *mirabilis* fitness within the bloodstream by conducting a co-challenge experiment of WT *P*. *mirabilis* and a non-motile *fliF* mutant (S6 Fig). As expected, the *fliF* mutant did not exhibit a significant competitive defect in the liver or the spleen, indicating that flagella do not contribute to *P*. *mirabilis* fitness in a direct bacteremia model. In conclusion, factors secreted through the twin arginine translocation system provide a significant fitness advantage to *P*. *mirabilis* during bacteremia that is independent of flagellum-mediated motility.

### Nitrogen assimilation is required for systemic infection

Acquisition of nitrogen is critical for bacterial production of amino and nucleic acids. In general, under conditions where the carbon to nitrogen ratio is low, the GS-GOGAT system (*glnA/gltB*) is utilized to incorporate ammonium via the production of two molecules of l-glutamate, and expression of *glnA* is activated by the two-component system NtrBC. Conversely, under conditions where the carbon to nitrogen ratio is high, glutamate dehydrogenase (*gdhA*) is favored for nitrogen assimilation through the addition of an amine group onto α-ketoglutarate to generate one molecule of l-glutamate. In the urinary tract, *P*. *mirabilis* appears to use one system or the other, but not both [28, 29]. It is therefore notable that *gdhA*, *glnA*, *gltB*, and *ntrB* were all identified as fitness factors for spleen colonization (fold-change 35.2, 19.4, 12.7, and 28.3, respectively) and *gltB* also had a defect in the liver (fold-change 4.4) (S5 Table). However, *glnA* was not an infection-specific fitness factor as it was also identified as having a defect during incubation in RPMI alone and in serum.

**Fig 8:**
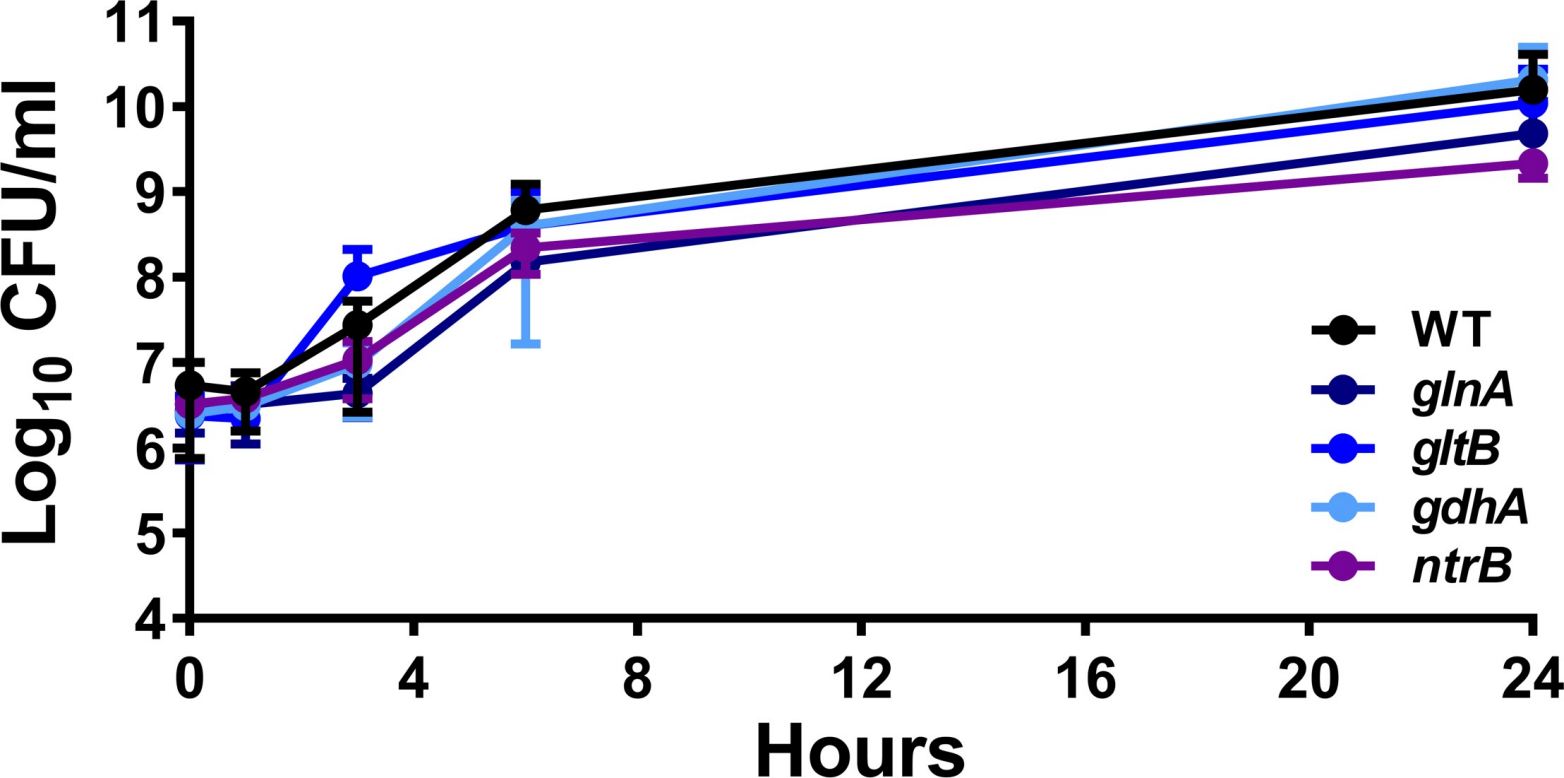
Nitrogen assimilation does not contribute to growth in serum *ex vivo*. 50% naive mouse serum was inoculated with 1x10^6^ CFU wild-type *P. mirabilis* or an isogenic mutant *glnA, gltB, gdhA*, or *ntrB*. Cultures were incubated statically at 37°C with 5% CO2 and sampled at 0, 1, 3, 6, and 24 hours for enumeration of CFUs on LB agar. Data for the *gltB* mutant are provided from Fig 4 for comparison. Error bars represent the mean ± standard deviation from three biological replicates.

To determine the contribution of these two pathways to growth and survival in the serum environment, we utilized mutants in all four nitrogen assimilation genes. Survival in mouse serum *in vitro* was first assessed as above, and none of the mutants exhibited growth defects in 50% naïve mouse serum (Fig 8). Growth in LB was next assessed to determine if any of the mutants exhibit defects in rich medium. Mutants in *gltB*, *gdhA*, and *ntrB* grew similarly to WT, and the *glnA* mutant exhibited a significant growth delay (*P*<0.001 by two-way ANOVA) but reached saturation comparable to WT by 18 hours (S7A Fig). When growth in PMSM minimal medium was assessed, the *gdhA* mutant exhibited a growth delay (*P*<0.009 by two-way ANOVA) but reached saturation comparable to WT, and loss of *glnA* resulted in glutamine auxotrophy, which could be fully complemented by the addition of 10 mM l-glutamine (S7B Fig). In RPMI, all mutants except for *ntrB* exhibited significant growth delays, and all except for *glnA* eventually reached saturation comparable to WT (S7C Fig).

**Fig 9:**
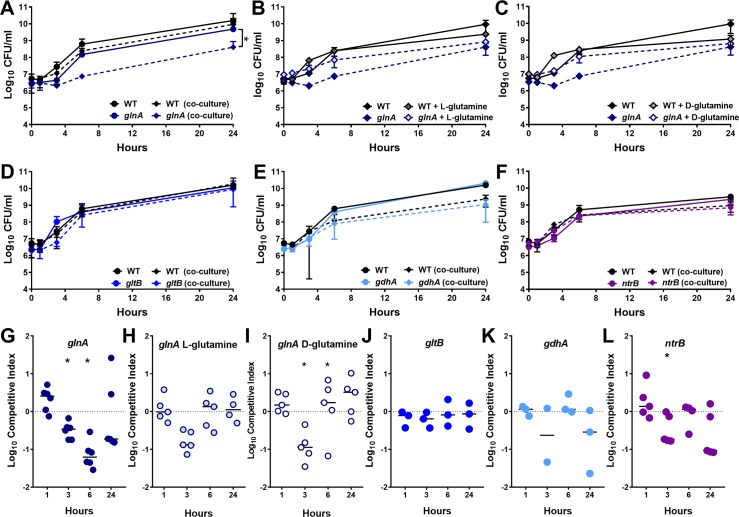
Nitrogen assimilation does not contribute to fitness in serum *ex vivo*. 50% naive mouse serum was inoculated with 1x10^6^ CFU wild-type *P. mirabilis*, an isogenic mutant, or a 1:1 mixture of wild-type *P. mirabilis* and isogenic mutant (co-culture). The following mutants that exhibited significant fitness defects in the liver and/or spleen during bacteremia were included: *glnA* (A), *gltB* (G), *gdhA* (I) and *ntrB* (K). Additionally, the growth of *glnA* in 50% mouse serum with or without supplementation of L-glutamine (C) or D-glutamine (E) was assayed. Cultures were incubated statically at 37°C with 5% CO2 and sampled at 0, 1, 3, 6, and 24 hours for enumeration of CFUs on LB agar with and without kanamycin. Error bars represent the mean ± standard deviation from three to five biological replicates. *P< 0.05 as determined by two-way ANOVA. (B, D, F, H, J, L) A competitive index (CI) was calculated for the mutant at each time point from the ratio of mutant to wild-type colonization level at the time indicated divided by the ratio of mutant to wild-type at time zero (see Materials and Methods). Each data point represents the CI from an individual replicate. Solid lines represent the median. Dashed lines indicate a competitive index of 1, or a 1:1 ratio of mutant to wild-type. Error bars represent the mean ± standard deviation from three to five biological replicates. *P< 0.05 as determined by Wilcoxon signed rank test.

To probe the relative contribution of the two nitrogen assimilation pathways to *P*. *mirabilis* survival in serum, each nitrogen mutant was cultured independently as well as co-cultured with the WT strain to assess fitness during incubation in naïve mouse serum (Fig 9A, 9G, 9I and 9K). A competitive index was calculated for the ratio of mutant to WT to determine fitness during co-culture (Fig 9B, 9H, 9J and 9L). Consistent with the Tn-Seq results, the *glnA* mutant exhibited a significant fitness defect during co-culture with WT *in vitro* (Fig 9A and 9B). Interestingly, this defect could be partially complemented by the addition of either l-glutamine (Fig 9C and 9D) or d-glutamine (Fig 9E and 9F), indicating that the defect is likely due to a combination of l-glutamine auxotrophy, defects in peptidoglycan biosynthesis (d-glutamine), and dysregulation of nitrogen assimilation. Loss of *gltB* or *gdhA* did not impact fitness in serum during co-culture *in vitro* (Fig 9G–9J), and loss of *ntrB* only resulted in a minor defect at 3 hours post-inoculation (Fig 9K and 9L). Thus, glutamine synthetase (*glnA*) is the only nitrogen assimilation factor that appears to contribute to fitness during growth in serum.

**Fig 10:**
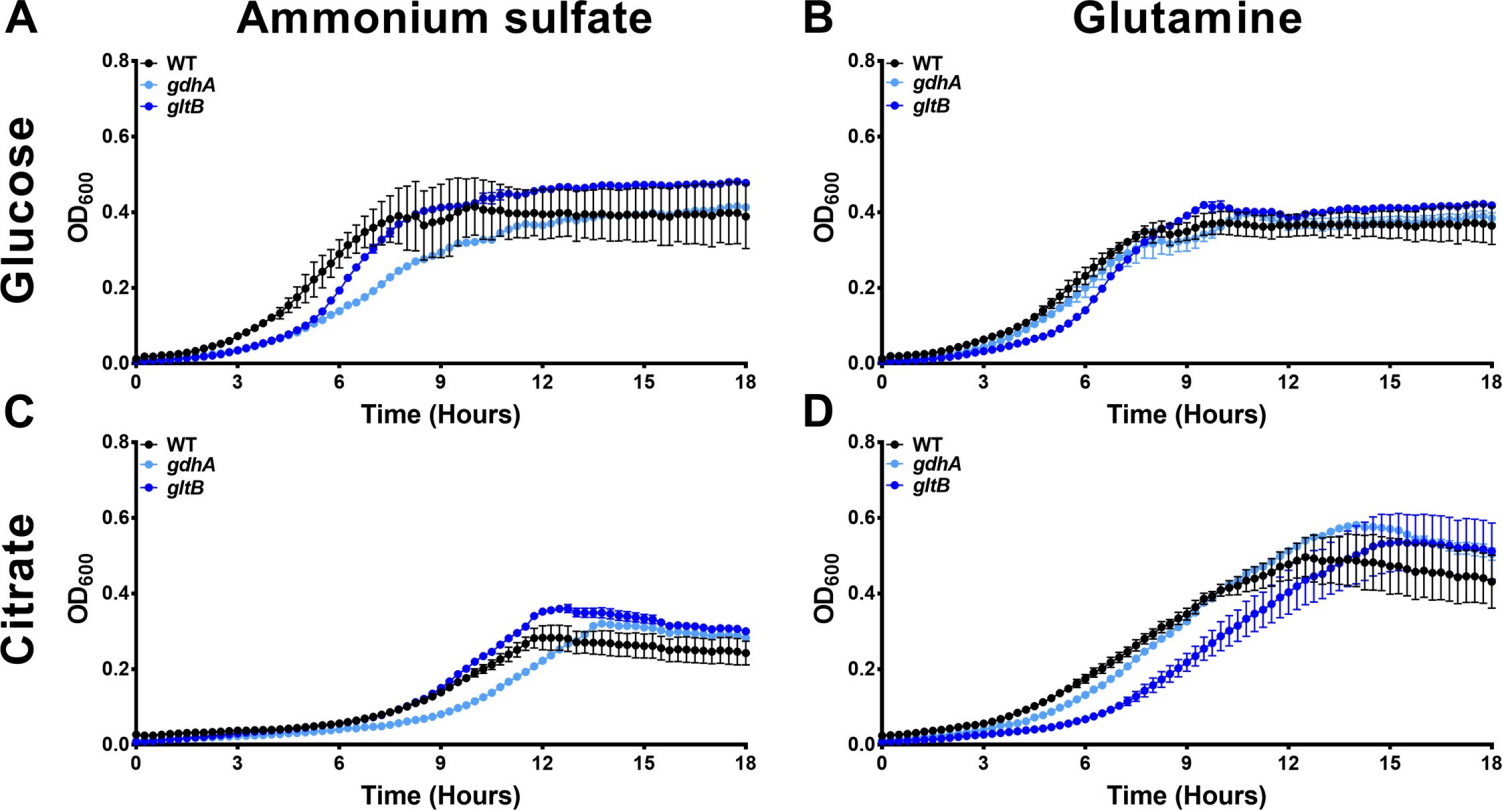
Different nitrogen assimilation pathways are required for optimal growth when carbon and nitrogen source are varied. Growth of wild-type *P. mirabilis* and isogenic mutants *gltB* and *gdhA* was measured by optical density at 600 nm every 15 min over the course of 18 hours during incubation at 37°C with shaking in either PMSM with glucose (A, B) or citrate (C, D) as the carbon source. Growth medium was supplemented 0.2% ammonium sulfate (A, C), or 0.2% ammonium sulfate with 0.2% glutamine (B, D). Error bars represent the mean ± standard deviation from three independent experiments, three replicates each.

Selected nitrogen mutants were next cultured in PMSM minimal medium with either glucose or citrate as a carbon source and either ammonium sulfate or l-glutamine as nitrogen sources (Fig 10). Glutamate dehydrogenase (*gdhA*) was required for optimal growth when ammonium was used as the nitrogen source regardless of carbon source (*P* = 0.020 for glucose and 0.019 for citrate by two-way ANOVA, Fig 10A and 10C), and the growth defect of the *gdhA* mutant was abrogated when l-glutamine was used as the nitrogen source (Fig 10B and 10D). The importance of the GS-GOGAT system was only assessed using *gltB*, to avoid potential confounding effects from glutamine auxotrophy in the *glnA* mutant. GOGAT mutants in other bacterial species exhibit growth defects in minimal medium when ammonium is used as the nitrogen source and glucose is used as the carbon source, and their growth defects can be alleviated by switching to a poor carbon source, such as citrate [40–42]. As expected, the *gltB* mutant exhibited a slight but significant growth defect when glucose was used as the carbon source independent of the nitrogen source (*P* = 0.029 for ammonium and *P* = 0.034 for glutamine, Fig 10A and 10B), and the defect during growth in ammonium was complemented when citrate was the carbon source (Fig 10C). However, citrate was unable to facilitate growth of the *gltB* mutant when glutamine was provided as the nitrogen source (*P* = 0.01 by two-way ANOVA, Fig 10D).

**Fig 11:**
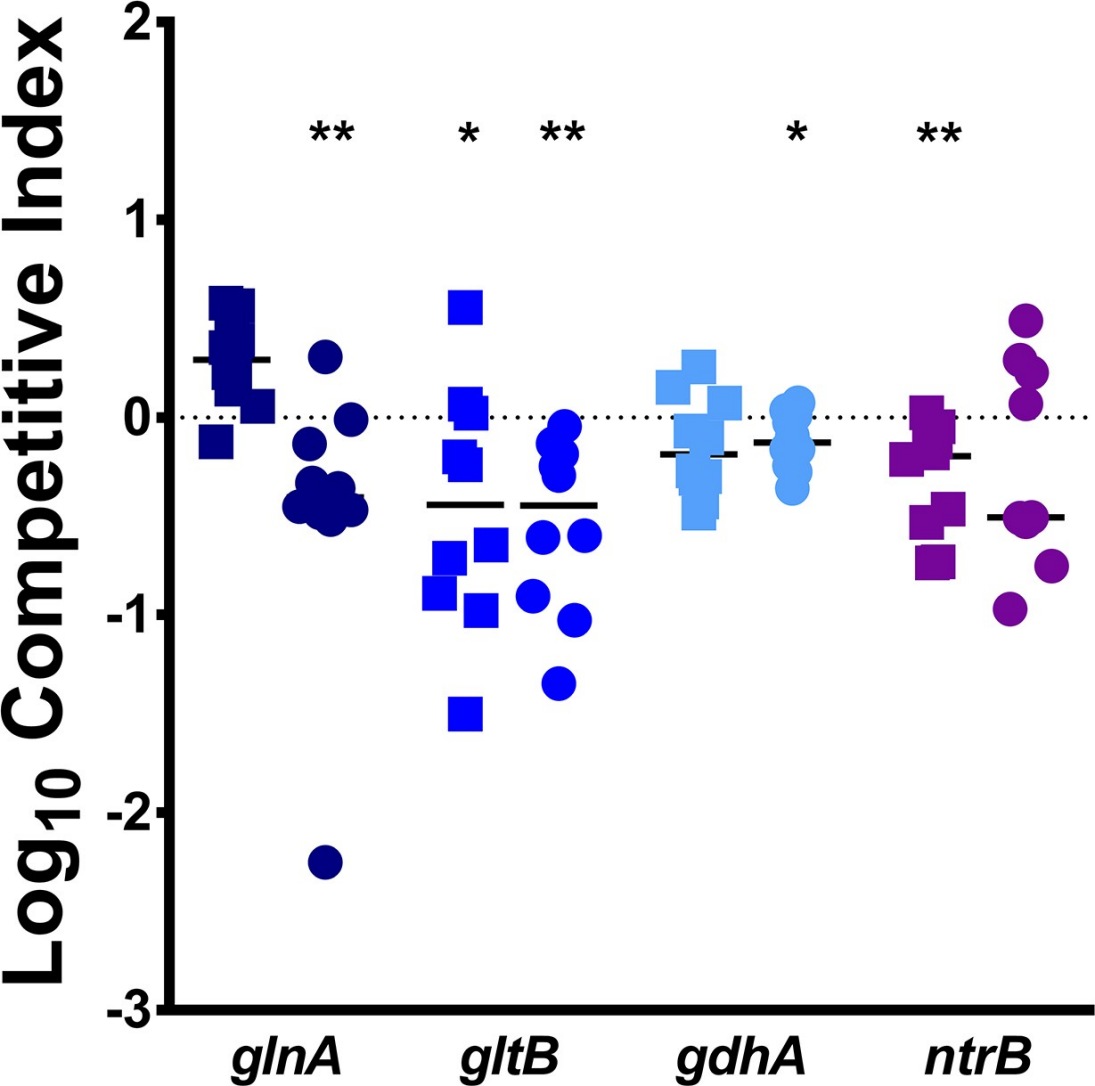
Nitrogen assimilation pathways contribute to fitness within the bloodstream. CBA/J mice were inoculated via the tail vein with 1x10^7^ CFU of a 1:1 mixture of wild-type *P. mirabilis* and an isogenic mutant. Liver and spleen were harvested from mice 24 hours post-inoculation, homogenized, and plated on LB agar and LB agar with kanamycin. A competitive index (CI) was calculated for each mutant on a per-mouse basis for the liver (square) and spleen (circle) as above. Data for the *gltB* mutant are provided from Fig 5 for comparison. Each data point represents the Log10 CI from an individual mouse. Solid lines represent the median. Dashed lines indicate a competitive index of 1, or a 1:1 ratio of mutant to wild-type. * P < 0.05 and ** P < 0.01 as determined by Wilcoxon signed rank test.

We next determined the contribution of the nitrogen assimilation pathways to fitness during murine co-challenge. All four mutants exhibited fitness defects *in vivo* in at least one organ (Fig 11). Notably, *glnA*, *gltB*, and *gdhA* all recapitulated the organ-specific defects predicted by the Tn-Seq results, while *ntrB* exhibited a defect in the liver but not the spleen, as fitness of the *ntrB* mutant appeared to follow a bimodal distribution in the spleen. The magnitude of the fitness defects for the nitrogen mutants *in vivo* in concert with the *in vitro* defects observed for these mutants indicate that GS-GOGAT is likely the critical pathway for nitrogen assimilation by *P*. *mirabilis* within the bloodstream. Thus, the bloodstream likely presents *P*. *mirabilis* with relatively low levels of ammonium and poor carbon sources. This is also in agreement with the fact that the concentration of urea in blood is much lower than that in urine, and that urease does not contribute to fitness in the bacteremia model.

### Polyamine biosynthesis is required for systemic infection

Polyamines are known to play a critical role in bacterial growth and have been implicated as contributing to cell wall biosynthesis, siderophore biosynthesis, motility, cell signaling, and acid resistance [43, 44]. Putrescine is a polyamine that can be produced in two ways in *P*. *mirabilis* HI4320: 1) the decarboxylation of L-arginine to agmatine by arginine decarboxylase (*speA*) followed by the conversion of agmatine into putrescine by agmatinase (*speB*); and 2) decarboxylation of ornithine to putrescine by ornithine decarboxylase (*speF*). Putrescine can also be imported via the spermidine/putrescine ABC transporter complex *potABCD*. All of these genes were identified as infection-specific fitness factors in the spleen (fold-change 7.3–59.8), and *speAB* also exhibited liver defects (fold-change 13.8 and 9.9, respectively, S5 Table), although only *speA* was determined to have a statistically significant liver defect. In addition to its function in putrescine synthesis, *speA* also contributes to maintenance of membrane potential during arginine catabolism, and both functions have been shown to contribute to colonization of the urinary tract [28, 44].

**Fig 12:**
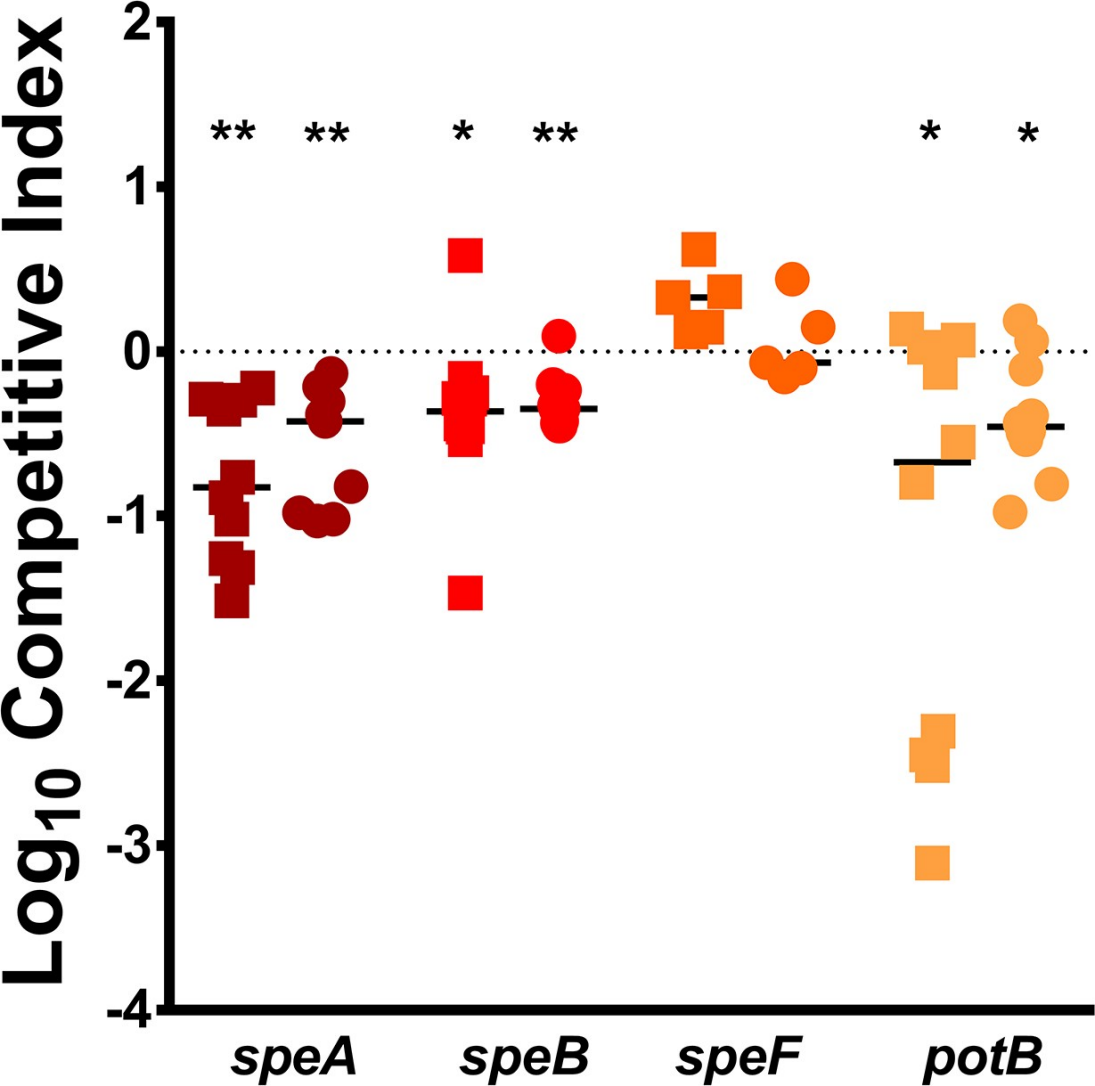
Polyamine biosynthesis contributes to fitness with the bloodstream. CBA/J mice were inoculated via the tail vein with 1x10^7^ CFU of a 1:1 mixture of wild-type *P. mirabilis* and an isogenic mutant. Liver and spleen were harvested from mice 24 hours post-inoculation, homogenized, and plated on LB agar and LB agar with kanamycin. A competitive index (CI) was calculated for each mutant on a per-mouse basis for the liver (square) and spleen (circle) as above. Data for the *speA* mutant are provided from Fig 5 for comparison. Each data point represents the Log10 CI from an individual mouse. Solid lines represent the median. Dashed lines indicate a competitive index of 1, or a 1:1 ratio of mutant to wild-type. * P < 0.05 and ** P < 0.01 as determined by Wilcoxon signed rank test.

**Fig 13:**
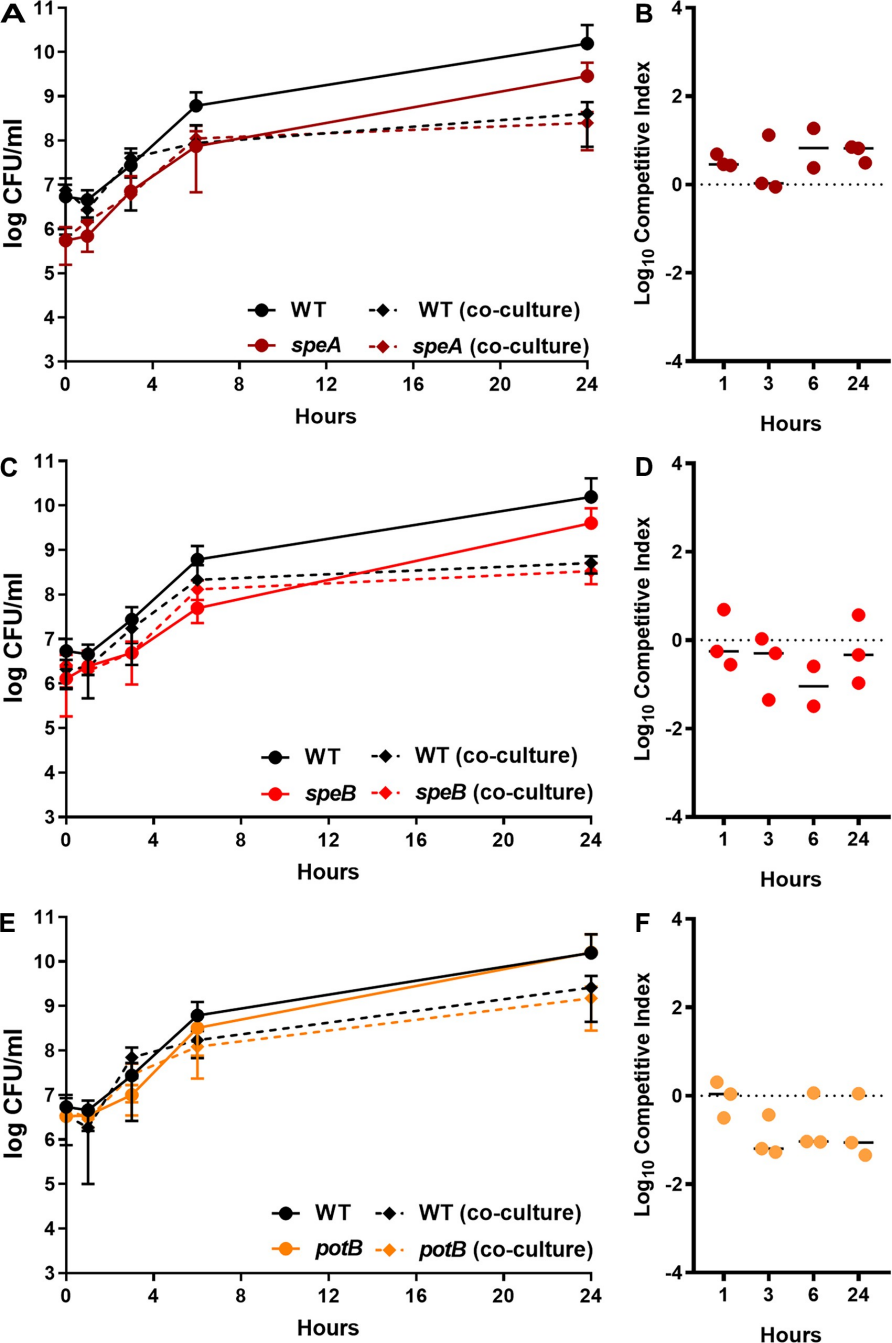
Polyamine biosynthesis is not required for fitness in serum *ex vivo*. 50% naive mouse serum was inoculated with 1x10^6^ CFU wild-type *P. mirabilis*, an isogenic mutant, or a 1:1 mixture of wild-type *P. mirabilis* and isogenic mutant (co-culture). The following mutants that exhibited significant fitness defects in the liver and/or spleen during bacteremia were included: *speA* (A), *speB* (C), and *potB* (E). Cultures were incubated statically at 37°C with 5% CO2 and sampled at 0, 1, 3, 6, and 24 hours for enumeration of CFUs on LB agar with and without kanamycin. Error bars represent the mean ± standard deviation from three biological replicates. (B, D, F) A competitive index (CI) was calculated for the mutant at each time point from the ratio of mutant to wild-type colonization level at the time indicated divided by the ratio of mutant to wild-type at time zero (see Materials and Methods). Each data point represents the CI from an individual replicate. Solid lines represent the median. Dashed lines indicate a competitive index of 1, or a 1:1 ratio of mutant to wild-type. Error bars represent the mean ± standard deviation from two or three biological replicates.

To explore the relative importance of arginine degradation and polyamine biosynthesis to fitness during bacteremia, we utilized mutants in *speA*, *speB*, *speF*, and *potB*. None of the mutants exhibited significant growth defects in LB broth (S8A Fig), and *speB* was comparable to *speA* in PMSM, exhibiting a significantly increased lag phase (*P*<0.001 by two-way ANOVA, S8B Fig). In RPMI, all four mutants exhibited increased lag phases, but were ultimately able to reach comparable density as WT at 18 hours (*P*<0.012 by two-way ANOVA, S8C Fig). We next challenged mice with wild-type HI4320 and each of the isogenic mutants as above. Disruption of *speA*, *speB*, or *potB* resulted in a fitness defect in the liver and spleen, indicating that putrescine biosynthesis and import are important for fitness during bacteremia (Fig 12). The *speF* mutant did not exhibit a defect, further indicating that *P*. *mirabilis* primarily uses the *speAB* pathway for putrescine biosynthesis, consistent with previous *in vitro* findings [45]. Notably, the repressor of the arginine biosynthetic pathway (*argR*) was also identified as an infection-specific fitness factor in the spleen (33.9 fold-change, S5 Table), supporting the hypothesis that putrescine biosynthesis is the more critical function of *speA* than arginine decarboxylation for maintenance of membrane potential. Importantly, the defects in colonization observed during co-challenge with wild-type HI4320 were infection-specific, as co-culture *in vitro* in 50% mouse serum did not result in differences in CFU of mutant compared to wild-type (Fig 13A–13F). Thus, putrescine import and biosynthesis likely represent infection-specific fitness factors for bacteremia.

## Discussion

*Proteus mirabilis* HI4320 has been used as a model strain for decades to explore virulence determinants of this unusual bacterial species, particularly for urinary tract infection [2, 5]. With the availability of the complete genome sequence in 2008 [30], *in vivo* transcriptome assessment [29], and signature-tagged mutagenesis studies [34, 46, 47], much has been learned concerning how this organism adapts to the urinary tract and the virulence factors that contribute to ascending UTI [see [5, 48, 49] for review]. Our prior study extended this work by detailing the use of Tn-Seq for identification of fitness factors in a murine model of catheter-associated urinary tract infection (CAUTI), which uncovered genes essential for growth in rich medium and numerous previously unrecognized *P*. *mirabilis* fitness determinants, while also highlighting key differences in fitness requirements during ascending UTI *versus* CAUTI [28]. Several *P*. *mirabilis* fitness factors identified through these studies have been tested for contribution to spleen colonization during secondary bacteremia, yet global fitness of *P*. *mirabilis* within the bloodstream has never been directly assessed. In the present study, we utilized Tn-Seq to elucidate fitness determinants for bloodstream infection *versus* factors that contribute to survival in serum *in vitro*. Through these efforts, we identified 143 candidate infection-specific fitness factors for survival of *P*. *mirabilis* within the bloodstream.

Nine genes of interest were chosen for an initial validation of the Tn-Seq results: 3 mutants were generated to validate candidate fitness factors for *in vitro* defects (*asnA*, *cvpA*, and *hflK*) and 6 for candidate infection-specific defects (*arnA*, *btuB*, *cutC*, *gltB*, *speA*, and *tatC*). Seven of the nine mutants (78%) recapitulated the expected *in vitro* results, and 3/6 (50%) recapitulated the expected *in vivo* fitness defects. Notably, the genes that failed to validate were either not contained within an operon (*btuB* and *cutC*), or were within a fairly large operon where only half of the genes were predicted to have fitness defects (*arnA*). BtuB and CutC were also the only genes pertaining to vitamin B12 transport and choline utilization that were identified as candidate infection-specific fitness factors. Taken together, these data may indicate that *arnA*, *btuB*, and *cutC* were false-positives, which could be due to a variety of reasons including the coverage of TA insertion sites within these genes and the fold-change and *P* value cutoffs used in the analysis. Alternatively, the failure of these mutants during *in vivo* validation could pertain to the specific gene location utilized for generation of the TargeTron mutants *versus* the distinct transposon insertion sites within the genes that exhibited the greatest defects in the initial screen. It is also possible that the mutants only exhibit significant defects when dramatically underrepresented in the input inoculum, such as the ~1:50,000 ratio that would be present during the Tn-Seq screen, but not during a 1:1 co-challenge.

It is also important to note the myriad of technical limitations of Tn-Seq studies that could result in false hits, and stress the importance of secondary validation of hits from the screen *in vivo* with clean deletions. One example is the potential under-representation of mutants in a particular gene within the library. The total number of copies of mutants that can be mapped to one gene will be dependent on the chromosomal location (those closer to the origin have a higher mutation rate due to more copies of sequence near the origin in rapidly replicating cells), length of the gene in comparison to number of TA sites (not all genes have the same number of possible locations for transposon insertion), and accessibility of the DNA to transposon mutagenesis (obstruction of the transposase due to three dimensional structure of the DNA). Interruption of a gene with a transposon may also result in a growth delay on LB agar, which would result in fewer copies of a particular mutant when the library is generated compared to those that do not impact growth. Another example of a technical limitation is that our transposon was not designed to prevent polar effects downstream of the insertion site, especially in the last 20% of the gene.

Despite these potential limitations, the total validation rate for all mutants assessed under *in vitro* and *in vivo* conditions in this study was 12/16 (75%), which is consistent with other bacteremia Tn-Seq studies (ranging from 75–86%) [35, 50–52]. An additional infection-specific gene (d-serine ammonia lyase, *dsdA*) was also validated as having a fitness defect in the direct bacteremia model in a separate study [53], bringing the total *in vivo* validation rate to 10/13 (77%). It is also notable that the input samples in this study identified 478 genes as essential for growth in rich medium due to lack of insertions, 436 (91%) of which were identified in our prior study [28]. Thus, while one experimental approach involved incubating a single pool of 50,000 mutants for 16 hours in LB broth and the other utilized 5 pools of 10,000 mutants each incubated separately, the same genes were consistently identified as having a lack of transposon insertions in the input samples.

Several of the infection-specific fitness factors identified in this study have been previously implicated as important for *P*. *mirabilis* pathogenesis. Tn-Seq of fitness factors in a mouse model of CAUTI identified 116 of our 143 bacteremia hits as contributing to colonization of the bladder and/or kidneys colonization [28], and eight of these genes were also identified as fitness factors during ascending UTI by signature-tagged mutagenesis [5]. All genes with defects as determined by Tn-Seq that have been previously implicated as having a role in pathogenesis in other studies are demarcated in each of the supplemental tables. Furthermore, transcriptome profiling in the ascending model identified 39 of the infection-specific bacteremia fitness factors as being upregulated during ascending UTI [29]. Altogether, 117 (82%) infection-specific fitness factors for bacteremia have also be implicated as having a role in ascending UTI and/or CAUTI in other genome-wide studies [28, 29, 34]. Thus, there may be a core set of fitness factors that *P*. *mirabilis* requires for optimal colonization and persistence in a mammalian host regardless of infection route.

In addition to the overlap between fitness factors for bacteremia and UTI/CAUTI, seven of the 143 infection-specific fitness factors identified in this screen were previously verified as contributing to secondary bacteremia during co-challenge with the WT strain: *gdhA*, *pta*, and *cysJ* for ascending UTI [29, 54, 55], and *arnA*, *glnA*, *lon*, and *argR* for CAUTI [28]. Two of these genes (*gdhA* and *glnA*) both exhibited fitness defects when directly tested in the bacteremia model, while *arnA* did not. ArnA may therefore play a greater role in fitness of *P*. *mirabilis* during dissemination from the kidneys to spleen, or for survival directly within the bloodstream that is not recapitulated by tissue-resident bacterial populations in the bacteremia model. Taken together, these results underscore the critical importance of directly assessing candidate fitness genes identified through Tn-Seq studies, and for considering the importance of the route of inoculation (*e*.*g*., direct inoculation into the bloodstream, or dissemination to the bloodstream from a secondary route).

Our study has shed light on metabolic pathways that contribute to *P*. *mirabilis* fitness during bacteremia, and highlights key differences between how *P*. *mirabilis* adapts to the bloodstream *versus* the urinary tract. For instance, the urease enzyme is a well-known and critical fitness factor of *P*. *mirabilis*, and provides the bacterium with a nitrogen-rich environment through the hydrolysis of urea to ammonium and carbon dioxide. However, in the bloodstream, the urea concentration is 100- to 1,000-fold lower than in the urinary tract [31, 32]. Furthermore, the *K*_M_ of *P*. *mirabilis* urease for urea is only 60 mM, and thus would not catalyze urea hydrolysis well at bloodstream urea concentrations [56]. Consequently, *P*. *mirabilis* does not require urease activity for fitness in the bloodstream, and it appears to view the bloodstream as a relatively low-nitrogen environment. Another difference pertains to the role of arginine decarboxylase (*speA*). In the urinary tract, *P*. *mirabilis* appears to predominantly utilize this enzyme for its contribution to tolerance of the mildly-acidic urinary tract and proton motive force [44], but the role of this gene during bloodstream infection is most likely its contribution to polyamine biosynthesis.

Our investigation of *P*. *mirabilis* fitness requirements during bloodstream infection also revealed an important role for glucose transport and glycolysis. Several members of the phosphotransferase system were identified as infection-specific fitness factors in the spleen including, *ptsG*, *ptsH*, *ptsI*, *treB*, *nagE*, PMI2226, PMI2982, and PMI3515. Several genes involved in glycolysis were also identified as infection-specific fitness factors (*ptsG*, *gnd*, *edd*, and *pykA*), and genes involved in pyruvate catabolism (*aceEF*) were identified as fitness factors in serum *ex vivo* and during bacteremia. A previous study in *Serratia marcescens* also identified *ptsI* as a fitness factor during bloodstream infection, indicating that glucose transport and metabolism may represent an important fitness factor for survival of other Gram-negative bacteria within the bloodstream [51]. Interestingly, glucose uptake and glycolysis also contribute to *P*. *mirabilis* fitness during ascending UTI [29, 57], and four genes involved in these pathways were identified as potential fitness factors during CAUTI [28]. Taken together, these results indicate that glucose transport and metabolism are critical for *P*. *mirabilis* pathogenesis during bacteremia, and may represent fitness factors for establishment of several different types of infection by this bacterium.

Twin arginine translocation (Tat) is another fitness factor for multiple types of *P*. *mirabilis* infection. In this study, we demonstrate the importance of the Tat system for fitness during bloodstream infection, in addition to previous implications of its involvement during CAUTI [28]. However, it remains to be determined which Tat substrates provide the greatest fitness advantage to *P*. *mirabilis* within each infection setting. In *E*. *coli*, deletion of *tatABC* results in a motility defect due to a complete lack of flagellin synthesis [38]. This is not the case for *tatA* or *tatC* mutants of *P*. *mirabilis*, as they retained flagella-mediated swarming motility despite having lost swimming motility. Furthermore, a *fliF* mutant that does not produce flagella and is non-motile [34] was able to colonize the liver and spleen to a similar level as WT *P*. *mirabilis*, indicating that the defects observed for the Tat mutants stem from loss of motility-independent secreted substrates, such as factors involved in metabolism.

Notably, the 143 infection-specific fitness factors identified in this screen are well represented among *Proteus* species isolates as well as other Gram-negative species that commonly cause bloodstream infections. Specifically, homologs to 14 of the 143 *P*. *mirabilis* infection-specific fitness factors were also identified as fitness factors for bacteremia in *Acinetobacter baumanii*, homologs to 8 were identified in *Serratia marcescens*, and homologs to 7 were identified in *Citrobacter freundii* [35, 51, 58]. The *acr* multidrug efflux system was a fitness factor for *P*. *mirabilis* bacteremia, and was also identified as contributing to bacteremia in *E*. *coli* and *S*. *marcescens* [51, 52]. Similarly, the iron-sulfur cluster transcriptional regulator *fur* was a fitness factor for bacteremia in *P*. *mirabilis* as well as *S*. *marcescens* and *C*. *freundii* [35, 51]. There were also several cases where a given gene may not be identified as a fitness factor between species, but other genes within that operon or pathway were identified as fitness factors in the other species. Thus, there are likely shared pathways between Gram-negative bacteria that contribute to survival within the bloodstream.

In summary, the use of Tn-Seq as a high-throughput screen has enabled us to investigate the importance of individual genes during *P*. *mirabilis* bacteremia, a serious and often fatal complication of CAUTI. By combining assessment of fitness factors for *in vivo* bacteremia and *ex vivo* serum survival, we have identified infection-specific fitness factors that contribute to *P*. *mirabilis* survival within the bloodstream. Considering that almost 50% of these bacteremia-specific fitness factors have also been implicated as contributing to fitness in the urinary tract, the combined knowledge gained through these studies may uncover core requirements of this multidrug-resistant bacterium for colonization and pathogenesis in a wide range of infection models. These factors would be ideal targets for prevention or treatment of *P*. *mirabilis*, particularly in vulnerable populations such as catheterized nursing home residents.

## Materials and methods

### Ethics statement

All animal protocols were approved by the Institutional Animal Care and Use Committee (IACUC) at the University of Michigan Medical School (PRO00007111) and State University of New York at Buffalo Jacobs School of Medicine and Biomedical Sciences (MIC31107Y), and in accordance with the Office of Laboratory Animal Welfare (OLAW), the United States Department of Agriculture (USDA), and the guidelines specified by the Association for Assessment and Accreditation of Laboratory Animal Care, International (AAALAC, Intl.). Mice were euthanized by inhalant anesthetic overdose (UM) or CO_2_ (UB) followed by vital organ removal.

### Bacterial strains and culture conditions

*Proteus mirabilis* HI4320 was isolated in a prior study from the urine of a catheterized patient in a chronic care facility in Baltimore, Maryland [2, 59]. The *P*. *mirabilis* HI4320 transposon mutant library was previously constructed, validated, and successfully utilized in a mouse model of CAUTI [28]. Bacteria were routinely cultured at 37°C with aeration in 5 ml LB broth (10 g/L tryptone, 5 g/L yeast extract, 0.5 g/L NaCl) or on LB solidified with 1.5% agar. *Proteus mirabilis* minimal salts medium (PMSM) (10.5 g/L K_2_HPO_4_, 4.5 g/L KH_2_PO_4_, 0.47 g/L sodium citrate, 1 g/L (NH_4_)_2_SO_4_, supplemented with 0.001% nicotinic acid, 1mM MgSO_4_, and 0.2% glycerol) [60] or RPMI with 2 mM L-glutamine (Sigma). Transposon mutants were cultured in LB containing 25 μg/ml kanamycin (Sigma). Additional *P*. *mirabilis* mutants for validation of candidate fitness factors were constructed by insertion of a kanamycin resistance cassette as previously described using the TargeTron system (Sigma), and are listed in S8 Table [61]. Resulting mutants were screened by kanamycin selection and PCR. All primers for generation and verification of mutants are provided in S9 Table.

### Identification of infection-specific fitness factors

A schematic of the Tn-Seq experimental setup is provided in S2 Fig. Ten mice were inoculated intravenously with the *P*. *mirabilis* pool of 50,000 transposon mutants to assess fitness factors for survival within the bloodstream, as measured by recovery from spleens and livers 24 hours post-inoculation (hpi). All 10 mice exhibited adequate spleen and liver colonization to be included in the final analysis (S3 Fig). Concurrently, the transposon mutant pool was also subjected to the following *in vitro* culture conditions, in duplicate, to allow for identification of infection-specific fitness factors: 1) RPMI medium, 2) RPMI with 50% heat-inactivated naïve mouse serum (generated from CBA/J mice), and 3) RPMI with 50% heat-inactivated acute-phase serum (generated from CBA/J mice 5 hours after intraperitoneal injection with heat-killed *P*. *mirabilis*). Heat-inactivation of serum was achieved by incubation at 56°C for one hour. Following inoculation, all cultures were incubated statically at 37°C with 5% CO_2_ for 24 hours.

### Mouse model of bacteremia

Infection studies were carried out as previously described [62]. For determination of inoculating dose, female CBA/J mice were (Envigo) were inoculated by tail vein injection with 100 μl *P*. *mirabilis* HI430 suspended in phosphate-buffered saline (PBS: 0.128 M NaCl, 0.0027 M KCl, pH 7.4) to 1x10^6^, 1x10^7^, or 1x10^8^ CFU/ml. Mice were euthanized 24 hours post-inoculation (hpi), and organs were harvested into 3 ml PBS. Tissues were homogenized using an Omni TH homogenizer (Omni International), and plated onto LB agar using an Autoplate 4000 spiral plater (Spiral Biotech) for enumeration of colonies using a QCount automated plate counter (Spiral Biotech). For bottleneck determination, mice were inoculated with 100 μl containing mixtures of *P*. *mirabilis* HI4320 and the *ureF* mutant (1x10^8^ CFU/ml). Mice were euthanized and bacterial burden was enumerated as above by plating on plain LB (total CFUs) and LB containing kanamycin (*ureF* CFUs). For the Tn-Seq screen, transposon mutant pools (1 ml volume) were thawed in 9 ml fresh LB with kanamycin and cultured at 37°C for no more than 10 hours. Cultures were centrifuged to pellet, resuspended in PBS, and adjusted to 1x10^8^ CFU/ml. Ten CBA/J mice were inoculated by tail vein injection (100 μl of 1x10^8^ CFU/ml for a total inoculum of 1x10^7^ CFU/mouse). Mice were euthanized 24 hours post-inoculation (hpi), livers and spleens were harvested into PBS, tissues were homogenized as above, and a 150 μl aliquot was removed and spiral plated for enumeration of colonies. The remaining homogenates were spread plated in their entirety, and colonies were collected, pelleted, and frozen for sequencing. For validation experiments, 5–10 mice were inoculated with a 1:1 mixture of *P*. *mirabilis* HI4320 and a mutant of interest, and livers and spleens were homogenized for enumeration of colonies as above. Where indicated, a competitive index (CI) was calculated as follows:

$$\frac{CI=Strain\;A\;output/Strain\;B\;output}{Strain\;A\;input/Strain\;B\;input}$$

Log_10_CI = 0 indicates that the ratio of the strains in the output is similar to the input, and neither strain had an advantage. Log_10_CI>0 indicates that strain A has a competitive advantage over strain B. Log_10_CI<0 indicates that strain B has a competitive advantage over strain A.

### Illumina sequencing

Sequencing was conducted as described previously [28]. Briefly, genomic DNA was isolated from *P*. *mirabilis* in the inputs, serum samples, and the livers and spleens of all mice by hexadecyltrimethyl ammonium bromide (CTAB) precipitation [63]. Samples were enriched for transposon insertion junctions as outlined by Goodman *et al*. [64]. TapeStation analysis was used to confirm concentration and purity, and samples were multiplexed and subjected to V4 single end 50 HiSeq-2500 High-Output sequencing as follows: 1) input samples and serum samples (2 replicates each) were multiplexed and sequenced on a single lane; 2) 10 spleen samples were multiplexed and sequenced on a single lane; and 3) 10 liver samples were multiplexed and sequenced on a single lane. Each lane was spiked with 15% bacteriophage φX DNA to overcome low-diversity sequences. Sequencing was performed at the University of Michigan DNA core facility. The raw sequencing reads are available through the Sequence Read Archive under Study SRP182137: *Proteus mirabilis* bacteremia TNSeq, and the barcodes associated with each unique sample are provided in S10 Table.

### Mapping of transposon insertion-sites

Mapping to the *P*. *mirabilis* HI4320 chromosome and plasmid sequences (NCBI accession numbers NC_010054 and NC_010555) [30] was conducted using a modification to the Goodman In-Seq pipeline [64] as previously described [28].

### Identification of *P*. *mirabilis* fitness factors for survival in mouse serum and within the bloodstream

Individual genes were only assessed for fitness contribution if the mean of the sum of insertion-site reads was >1000 and the number of insertions in that gene was >5, to reduce potential over-estimation of fitness factors. The fitness contribution of each gene was then estimated as previously described [28] using an R package called TnseqDiff [65], which can be installed from the Comprehensive R Archive Network (CRAN). Significant genes for further analysis were selected based on an adjusted *P*-value <0.05 and >2-fold ratio of output over input.

### Proteome analysis

To identify homologs of infection-specific fitness factors first, a file was generated containing the FASTA sequences of each fitness factor, then the proteomes of other *P*. *mirabilis* strains available on PATRIC were compared to this sequence using the PATRIC proteome comparison tool [66]. For this comparison the sequence identity was limited to ≥10% over a minimum of 30% sequence.

### Growth curves

Overnight cultures of *P*. *mirabilis* mutants were washed once in PBS and diluted 1:100 in growth medium. Where indicated, PMSM was supplemented with 10 mg/mL of l-glutamine, d-glutamine, or l-asparagine. Carbon and nitrogen sources in PMSM were also adjusted as follows: carbon sources (0.2% glycerol, 0.2% citrate, or 0.2% glucose), nitrogen sources (0.002% or 0.2% ammonium sulfate or l-glutamine). A BioTek Synergy H1 96-well plate reader was utilized to generate growth curves. Cultures were incubated at 37°C with continuous shaking, and OD_600_ readings were taken every 15 min for 18 h. Serum growth curves were performed by inoculating 50% naïve mouse serum (Innovative Research) with 1x10^6^ CFU of PBS washed, overnight cultures of either an individual mutant or a 1:1 mixture of wild-type *P*. *mirabilis* and a mutant. Inoculated serum was incubated statically at 37°C with 5% CO_2._ Aliquots were taken at the time of inoculation and at indicated timepoints, diluted, and plated onto LB agar with and without kanamycin to determine CFUs of mutant and wild-type. Competitive indices for growth in serum were calculated as described above for murine infection studies.

### Tat substrate prediction

The Tat-substrate prediction software TatP [37] was used to identify Tat motifs and probable cleavage sites using the *P*. *mirabilis* HI4320 chromosome sequence (NCBI accession number NC_010054) [30].

### Motility assays

Swimming motility agar plates (MOT: 10 g/L tryptone, 0.5 g/L NaCl, 3 g/L agar) were stab-inoculated with an overnight culture of *P*. *mirabilis* HI4320 or isogenic mutant. MOT plates were incubated without inverting at 30°C for 18 hours prior to measurement of swimming diameter. Swarm agar refers to LB agar containing 5 g/L NaCl, and swarming was assessed by inoculating 5 μl of an overnight culture of *P*. *mirabilis* HI4320 or isogenic mutant onto the surface of a swarm plate, allowing the inoculum to soak in for ~10 minutes, and incubating at 37°C for 18 hours prior to measurement of the diameter of each swarm ring.

### Statistical analysis

Significance was assessed using Student’s t-test, two-way analysis of variance (ANOVA) with post-hoc multiple comparisons test, and Wilcoxon signed-rank test. These analyses were performed using GraphPad Prism, version 7 (GraphPad Software, San Diego, CA). All *P* values are two tailed at a 95% confidence interval.

## Supporting information

S1 Fig Determination of appropriate transposon library size for *P. mirabilis* bacteremia Tn-Seq. A) To determine the appropriate infectious dose of *P. mirabilis* HI4320 to achieve bacteremia, 3 CBA/J mice were inoculated via tail vein injection with 1x10^5^ CFU (light blue), 1x10^6^ CFU (medium blue), or 1x10^7^ CFU (dark blue) of an overnight culture of wild-type *P. mirabilis* HI4320. At 24 hours post inoculation, blood was collected and mice were sacrificed. The heart, lungs, kidneys, spleen, liver and brain were homogenized and plated on LB agar to determine bacterial burden. An inoculum containing 1x10^7^ CFU was determined to be ideal for assessment of *P. mirabilis* bacteremia. B) To verify that a kanamycin-resistant urease mutant (*ureF*) could be used to assess potential bottlenecks during bacteremia, CBA/J mice were infected via the tail vein with 1x10^7^ CFU of wild-type (n = 8) or 1x10^7^ CFU of the *ureF* mutant (n = 8). At 24 hours post inoculation, blood was collected and mice were sacrificed. Heart, lungs, kidneys, spleen, liver, and brain were homogenized and plated on LB agar to quantify CFUs. No significant differences in colonization were observed, indicating that the ureF mutant is suitable for bottleneck assessment. C) Colonization levels of liver (L), spleen (S), and kidneys (K) were determined 24 hours post-inoculation with 1x10^7^ CFU of a mixture of *ureF* and wild-type *P. mirabilis* at a ratio of 1:1, 1:1,000 or 1:10,000. The colonization level of the spleen and liver indicate that a library of less than 100,000 mutants could be used for Tn-Seq assessment, while kidney colonization is too low to support a mutant library larger than 1,000 mutants. D) To investigate possible bottlenecks, the ratio of *ureF* to wild-type was determined for the experiment presented in panel C by plating on LB agar and LB agar containing kanamycin. A competitive index (CI) was calculated using the ratio of mutant to wild-type in each organ divided by the ratio of mutant to wild-type from the inoculum. Dashed lines indicate a competitive index of +/- 100, or the range within which the ratio of mutant to wild-type is indicative of lack of a bottleneck in the bacteremia model. Bars represent the median and each dot represents a single mouse. A and B, dashed lines represent the limit of detection.
(TIF)

S2 Fig Conceptual model of *P. mirabilis* bacteremia TnSeq. A pool of 5x10^4^ transposon mutants was incubated at 37°C for no more than 10 hours and used to inoculate RPMI (output 1), 50% heat-inactivated naive mouse serum in RPMI (output 2), 50% heat-inactivated acute phase mouse serum generated from mice inoculated with heat killed *P. mirabilis* HI4320 (output 3) and 10 CBA/J mice via tail vein (spleen and liver outputs). In vitro, transposon pools were incubated statically at 37°C with 5% CO2 for 24 hours prior to plating on LB with kanamycin. In vivo, mice were sacrificed at 24 hours post-inoculation, and livers and spleens were homogenized and plated on LB with kanamycin. All output samples were generated in parallel to utilize the same input inoculum. Input and output pools of mutants were enriched for transposon insertion junctions and subjected to next generation Illumina sequencing. The resulting reads were mapped to the *P. mirabilis* HI4320 genome to determine both the location of the insertion and the abundance of each transposon mutant within the population.
(TIF)

S3 Fig Colonization levels of mice inoculated with transposon mutant pools. CBA/J mice were inoculated with 1x10^7^ CFU of a pool of 5x10^4^ transposon mutants thawed and incubated at 37°C for no more than 10 hours prior to inoculation. Mice were sacrificed 24 hours post-inoculation and bacterial burden per gram of tissue was determined by plating liver and spleen homogenates on LB with kanamycin. Each dot represents a single mouse, and bars represent the median. Dashed line indicates the limit of detection.
(TIF)

S4 Fig Growth of candidate fitness factor mutants in LB, PMSM, and RPMI. Growth of wild-type HI4320 and mutants was measured by optical density at 600 nm every 15 min over the course of 18 hours during incubation at 37°C with shaking in either LB (A, B), PMSM minimal medium (C, D) or RPMI (E, F). In addition, *asnA* was chemically complemented during growth in PMSM with 10 mM of L-asparagine. Error bars represent the mean ± standard deviation from two independent experiments, four replicates each.
(TIF)

S5 Fig Growth of Tat mutants in LB, PMSM, RPMI, and serum. Growth of wild-type HI4320, *tatA* and *tatC* strains was measured by optical density at 600 nm every 15 min over the course of 18 hours during incubation at 37°C with shaking in either LB (A), PMSM minimal medium (B), or RPMI (C). Error bars represent the mean ± standard deviation from two independent experiments, four replicates each. (D) Growth of wild-type HI4320, *tatA* and *tatC* strains over the course of 24 hours during static incubation at 37°C with 5% CO2 in 50% naive mouse serum. Cultures were sampled at 0, 1, 3, 6, and 24 hours for enumeration of CFUs on LB agar. Error bars represent the mean ± standard deviation from three replicates.
(TIF)

S6 Fig Competitive fitness of a *fliF* mutant during bacteremia. CBA/J mice were inoculated via the tail vein with 1x10^7^ CFU of a 1:1 mixture of wild-type *P. mirabilis* and an isogenic mutant. Liver and spleen were harvested from mice 24 hours post-inoculation, homogenized, and plated on LB agar and LB agar with kanamycin. A competitive index (CI) was calculated for each mutant on a per-mouse basis for the liver (square) and spleen (circle) as described above. Each data point represents the Log10 CI from an individual mouse. Solid lines represent the median. Dashed lines indicate a competitive index of 1, or a 1:1 ratio of mutant to wild-type. Determined non-significant (P>0.05) by Wilcoxon signed rank test.
(TIF)

S7 Fig Growth of nitrogen assimilation mutants in LB, PMSM, and RPMI. Growth of wild-type HI4320, *glnA, gltB, ghdA*, and *ntrB* strains was measured by optical density at 600 nm every 15 min over the course of 18 hours during incubation at 37°C with shaking in either LB (A), PMSM minimal medium (B) or RPMI (C). In addition, *glnA* was chemically complemented during growth in PMSM with 10 mM of L-glutamine. Error bars represent the mean ± standard deviation from two independent experiments, four replicates each.
(TIF)

S8 Fig Growth of polyamine biosynthesis and import mutants in LB, PMSM, and RPMI. Growth of wild-type HI4320, *speA, speB, speF*, and *potB* strains was measured by optical density at 600 nm every 15 min over the course of 18 hours during incubation at 37°C with shaking in either LB (A), PMSM minimal medium (B) or RPMI (C). Error bars represent the mean ± standard deviation from two independent experiments, four replicates each.
(TIF)

S1 Table Full dataset with *in vitro* and *in vivo* Tn-Seq results. (XLS)

S2 Table Candidate fitness factors for survival in RPMI. (XLSX)

S3 Table Candidate fitness factors for survival in mouse serum *ex vivo*. (XLSX)

S4 Table Serum-specific fitness factors. (XLSX)

S5 Table Candidate fitness factors for bacteremia. (XLSX)

S6 Table Homology of infection-specific fitness factors between *P. mirabilis* isolates. (XLSX)

S7 Table Predicted Tat substrates. (XLSX)

S8 Table Bacterial strains used in this study. (DOCX)

S9 Table Primers used in this study. (DOCX)

S10 Table Barcodes for deconvolution of Illumina sequence reads. (DOCX)
