# Supplementary figures and images for: Twin arginine translocation, ammonia incorporation, and polyamine biosynthesis are crucial for Proteus mirabilis fitness during bloodstream infection
Source: PLoS Pathog. 2019 Apr 22;15(4):e1007653. doi: 10.1371/journal.ppat.1007653 (PMC6497324; doi:10.1371/journal.ppat.1007653)

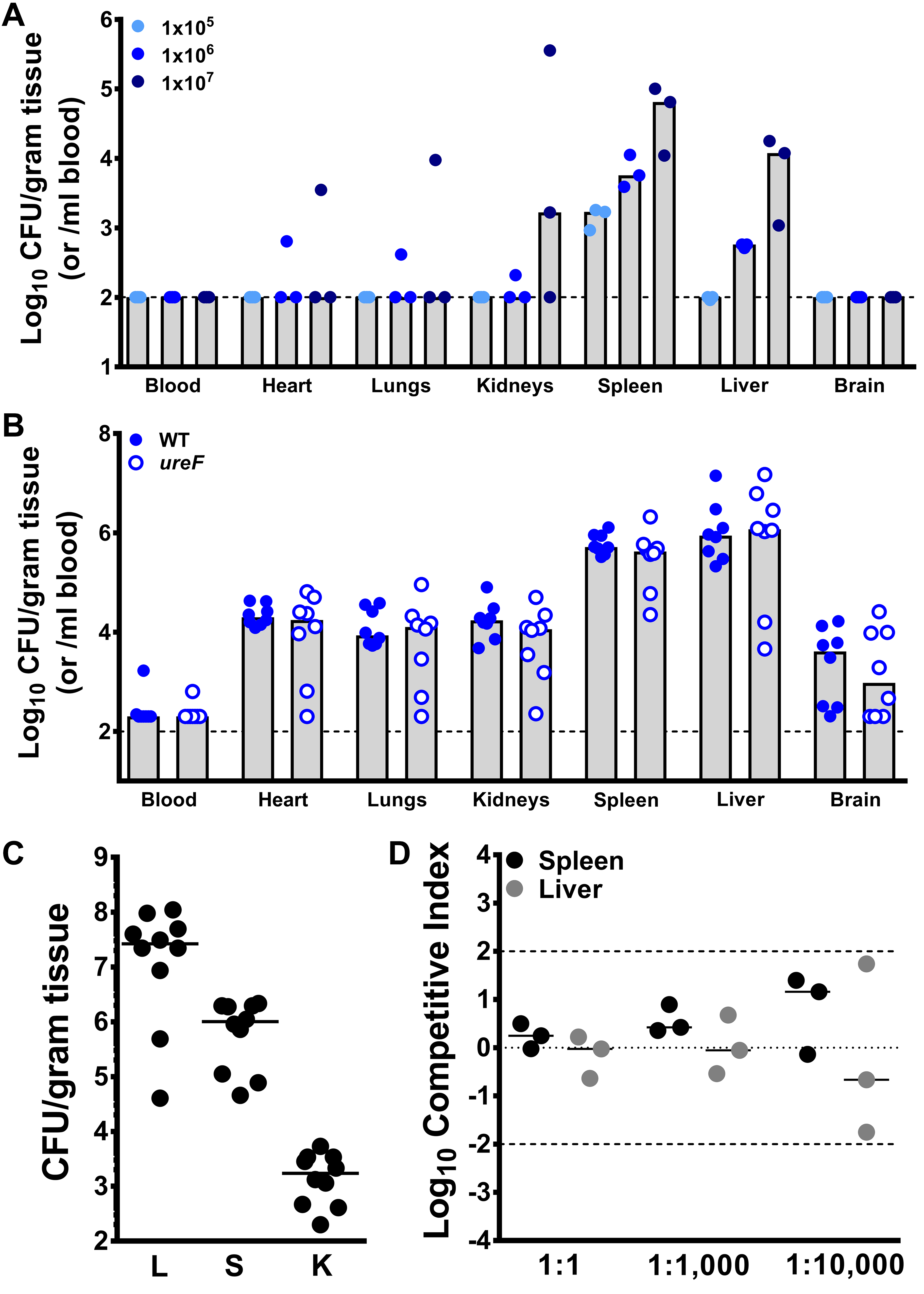

Supplement: S1 Fig — A) To determine the appropriate infectious dose of P. mirabilis HI4320 to achieve bacteremia, 3 CBA/J mice were inoculated via tail vein injection with 1x10^5^ CFU (light blue), 1x10^6^ CFU (medium blue), or 1x10^7^ CFU (dark blue) of an overnight culture of wild-type P. mirabilis HI4320. At 24 hours post inoculation, blood was collected and mice were sacrificed. The heart, lungs, kidneys, spleen, liver and brain were homogenized and plated on LB agar to determine bacterial burden. An inoculum containing 1x10^7^ CFU was determined to be ideal for assessment of P. mirabilis bacteremia. B) To verify that a kanamycin-resistant urease mutant (ureF) could be used to assess potential bottlenecks during bacteremia, CBA/J mice were infected via the tail vein with 1x10^7^ CFU of wild-type (n = 8) or 1x10^7^ CFU of the ureF mutant (n = 8). At 24 hours post inoculation, blood was collected and mice were sacrificed. Heart, lungs, kidneys, spleen, liver, and brain were homogenized and plated on LB agar to quantify CFUs. No significant differences in colonization were observed, indicating that the ureF mutant is suitable for bottleneck assessment. C) Colonization levels of liver (L), spleen (S), and kidneys (K) were determined 24 hours post-inoculation with 1x10^7^ CFU of a mixture of ureF and wild-type P. mirabilis at a ratio of 1:1, 1:1,000 or 1:10,000. The colonization level of the spleen and liver indicate that a library of less than 100,000 mutants could be used for Tn-Seq assessment, while kidney colonization is too low to support a mutant library larger than 1,000 mutants. D) To investigate possible bottlenecks, the ratio of ureF to wild-type was determined for the experiment presented in panel C by plating on LB agar and LB agar containing kanamycin. A competitive index (CI) was calculated using the ratio of mutant to wild-type in each organ divided by the ratio of mutant to wild-type from the inoculum. Dashed lines indicate a competitive index of +/- 100, or the range within which the [file ppat.1007653.s001.tif]

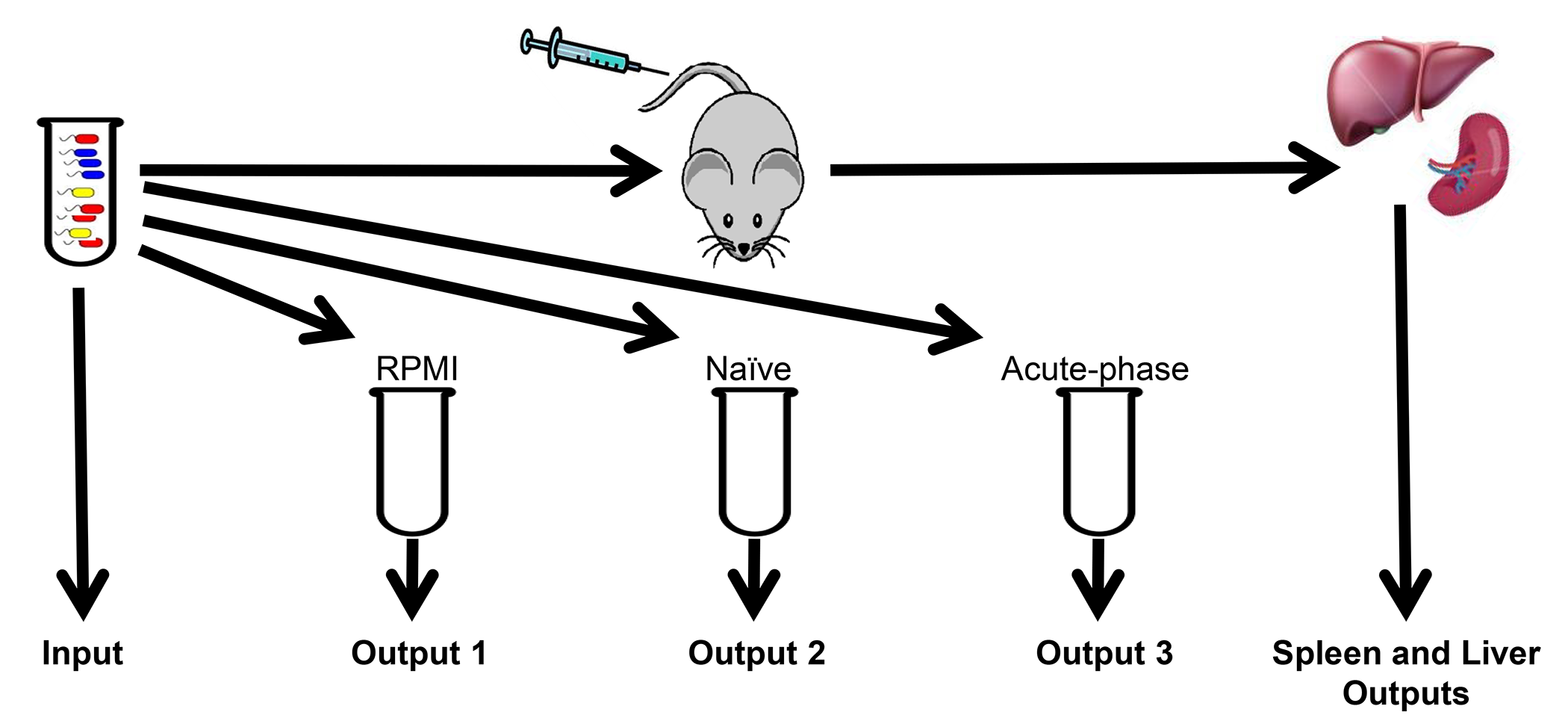

Supplement: S2 Fig — A pool of 5x10^4^ transposon mutants was incubated at 37°C for no more than 10 hours and used to inoculate RPMI (output 1), 50% heat-inactivated naive mouse serum in RPMI (output 2), 50% heat-inactivated acute phase mouse serum generated from mice inoculated with heat killed P. mirabilis HI4320 (output 3) and 10 CBA/J mice via tail vein (spleen and liver outputs). In vitro, transposon pools were incubated statically at 37°C with 5% CO2 for 24 hours prior to plating on LB with kanamycin. In vivo, mice were sacrificed at 24 hours post-inoculation, and livers and spleens were homogenized and plated on LB with kanamycin. All output samples were generated in parallel to utilize the same input inoculum. Input and output pools of mutants were enriched for transposon insertion junctions and subjected to next generation Illumina sequencing. The resulting reads were mapped to the P. mirabilis HI4320 genome to determine both the location of the insertion and the abundance of each transposon mutant within the population. (TIF) [file ppat.1007653.s002.tif]

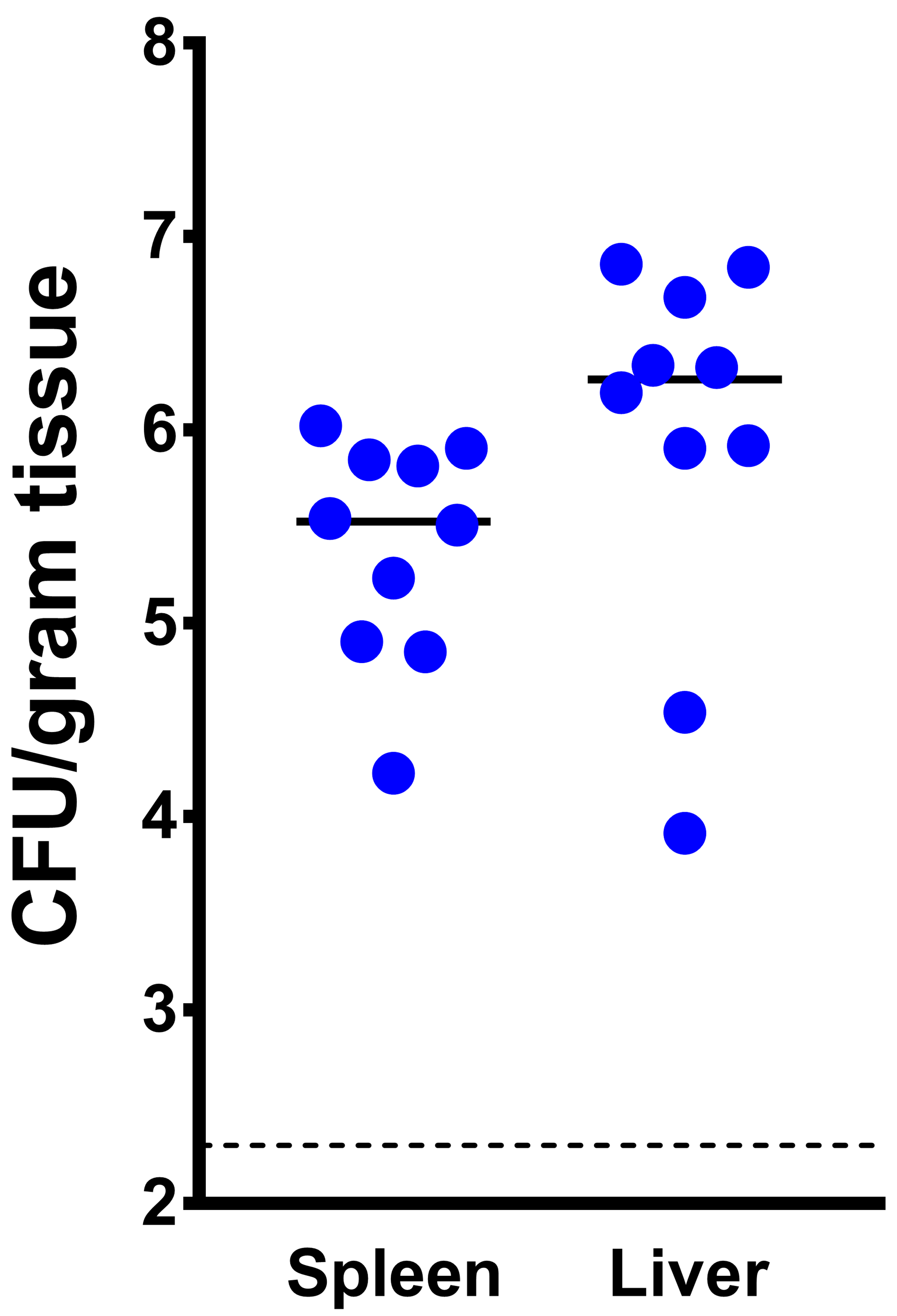

Supplement: S3 Fig — CBA/J mice were inoculated with 1x10^7^ CFU of a pool of 5x10^4^ transposon mutants thawed and incubated at 37°C for no more than 10 hours prior to inoculation. Mice were sacrificed 24 hours post-inoculation and bacterial burden per gram of tissue was determined by plating liver and spleen homogenates on LB with kanamycin. Each dot represents a single mouse, and bars represent the median. Dashed line indicates the limit of detection. (TIF) [file ppat.1007653.s003.tif]

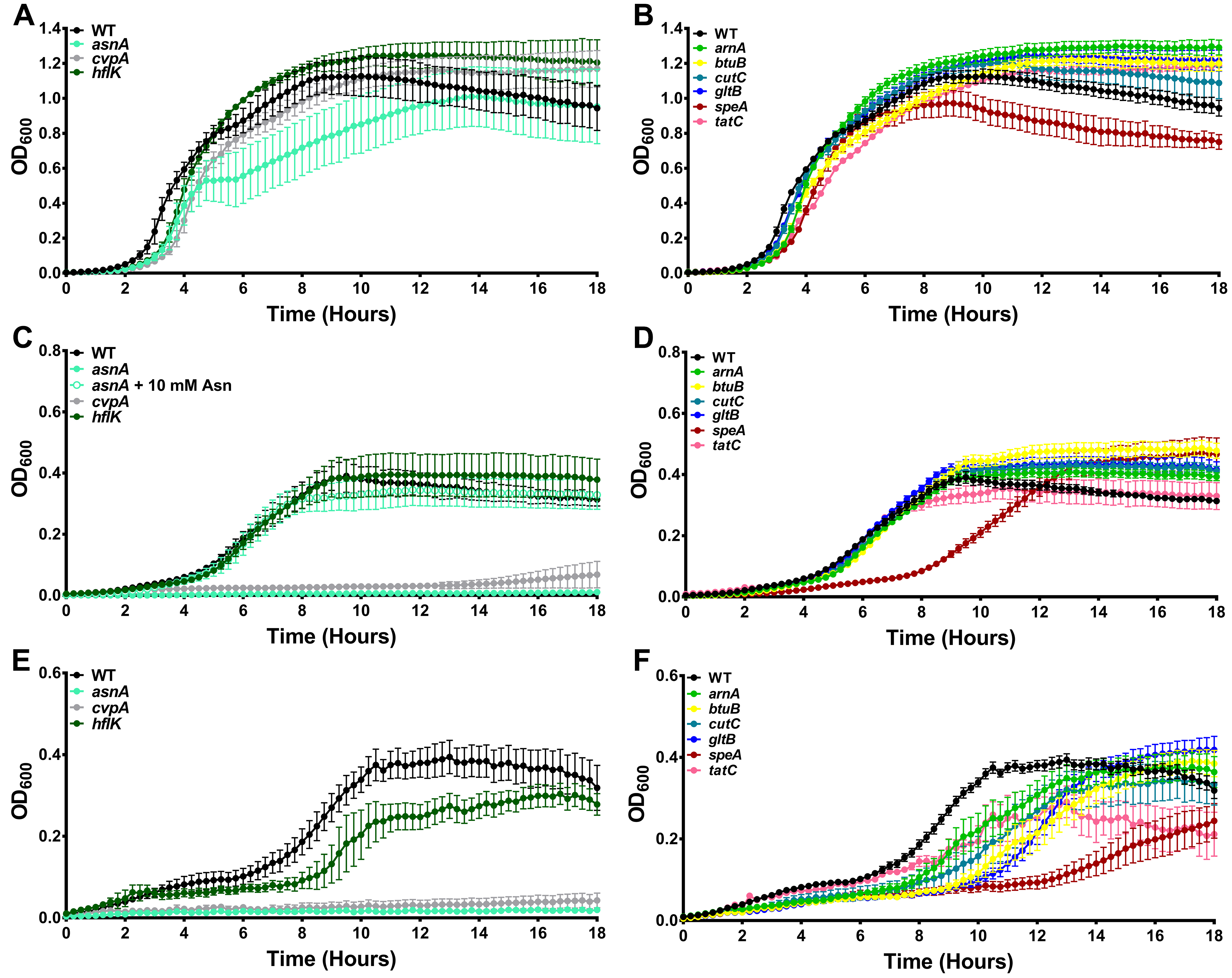

Supplement: S4 Fig — Growth of wild-type HI4320 and mutants was measured by optical density at 600 nm every 15 min over the course of 18 hours during incubation at 37°C with shaking in either LB (A, B), PMSM minimal medium (C, D) or RPMI (E, F). In addition, asnA was chemically complemented during growth in PMSM with 10 mM of L-asparagine. Error bars represent the mean ± standard deviation from two independent experiments, four replicates each. (TIF) [file ppat.1007653.s004.tif]

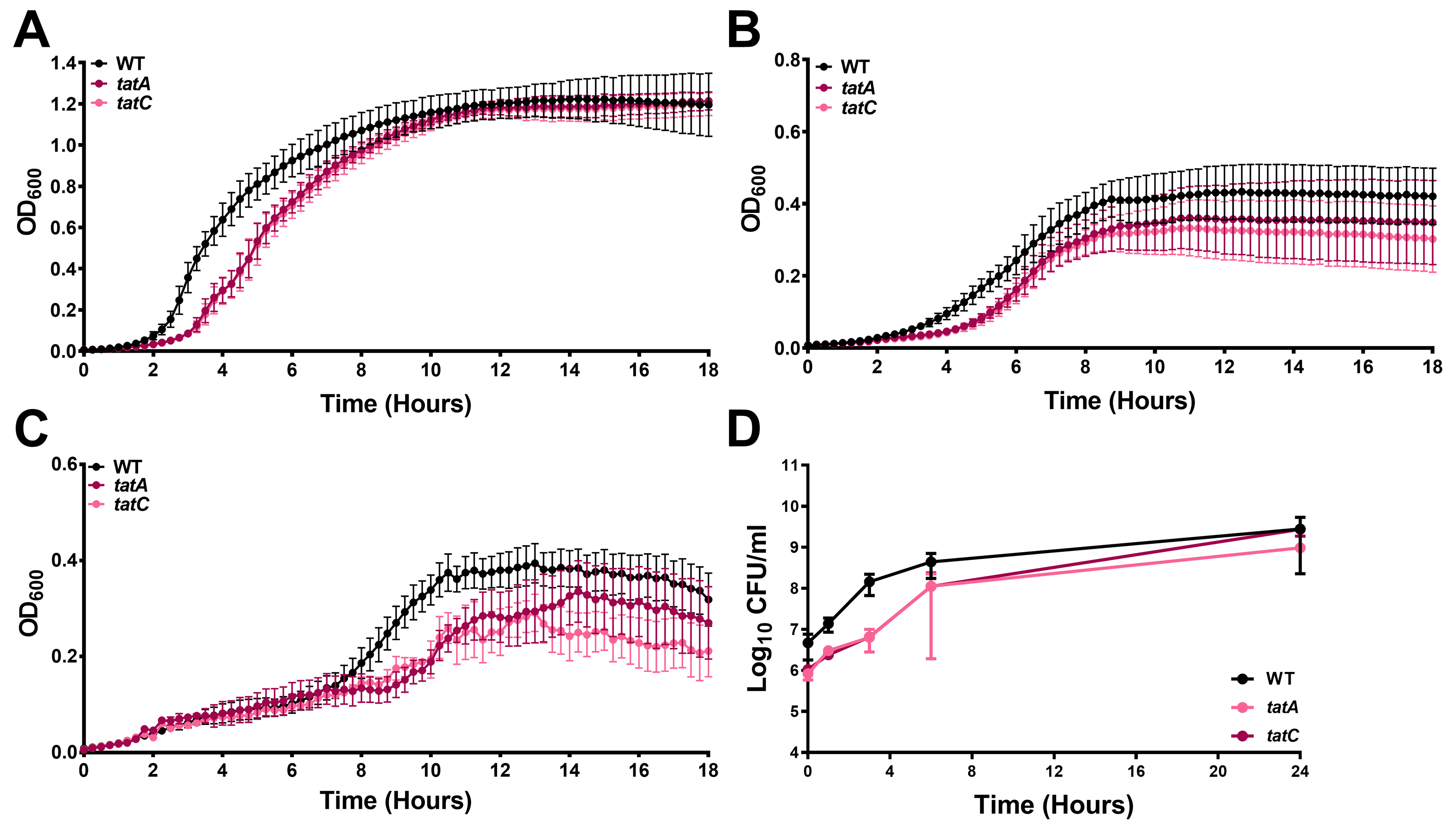

Supplement: S5 Fig — Growth of wild-type HI4320, tatA and tatC strains was measured by optical density at 600 nm every 15 min over the course of 18 hours during incubation at 37°C with shaking in either LB (A), PMSM minimal medium (B), or RPMI (C). Error bars represent the mean ± standard deviation from two independent experiments, four replicates each. (D) Growth of wild-type HI4320, tatA and tatC strains over the course of 24 hours during static incubation at 37°C with 5% CO2 in 50% naive mouse serum. Cultures were sampled at 0, 1, 3, 6, and 24 hours for enumeration of CFUs on LB agar. Error bars represent the mean ± standard deviation from three replicates. (TIF) [file ppat.1007653.s005.tif]

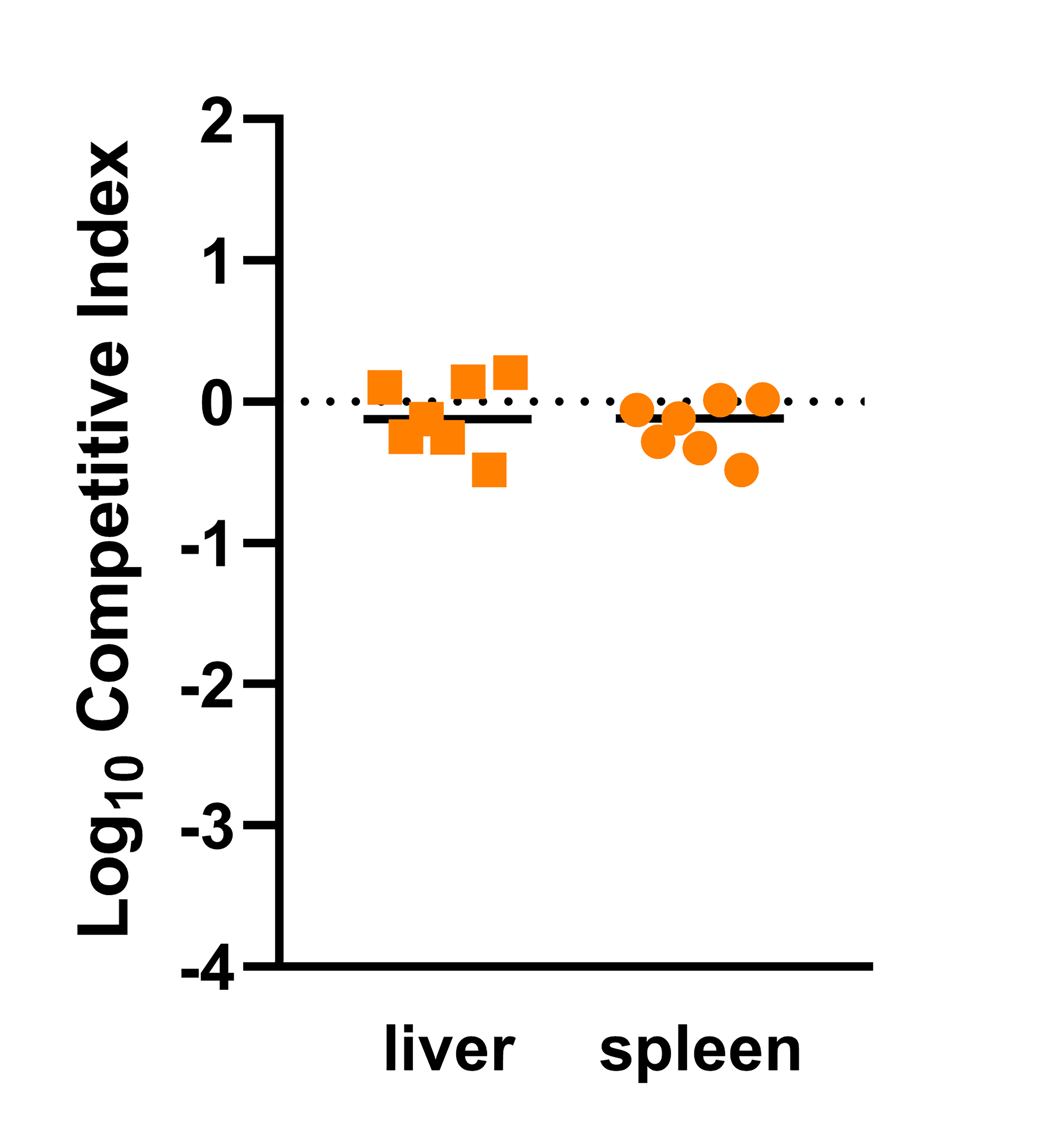

Supplement: S6 Fig — CBA/J mice were inoculated via the tail vein with 1x10^7^ CFU of a 1:1 mixture of wild-type P. mirabilis and an isogenic mutant. Liver and spleen were harvested from mice 24 hours post-inoculation, homogenized, and plated on LB agar and LB agar with kanamycin. A competitive index (CI) was calculated for each mutant on a per-mouse basis for the liver (square) and spleen (circle) as described above. Each data point represents the Log10 CI from an individual mouse. Solid lines represent the median. Dashed lines indicate a competitive index of 1, or a 1:1 ratio of mutant to wild-type. Determined non-significant (P>0.05) by Wilcoxon signed rank test. (TIF) [file ppat.1007653.s006.tif]

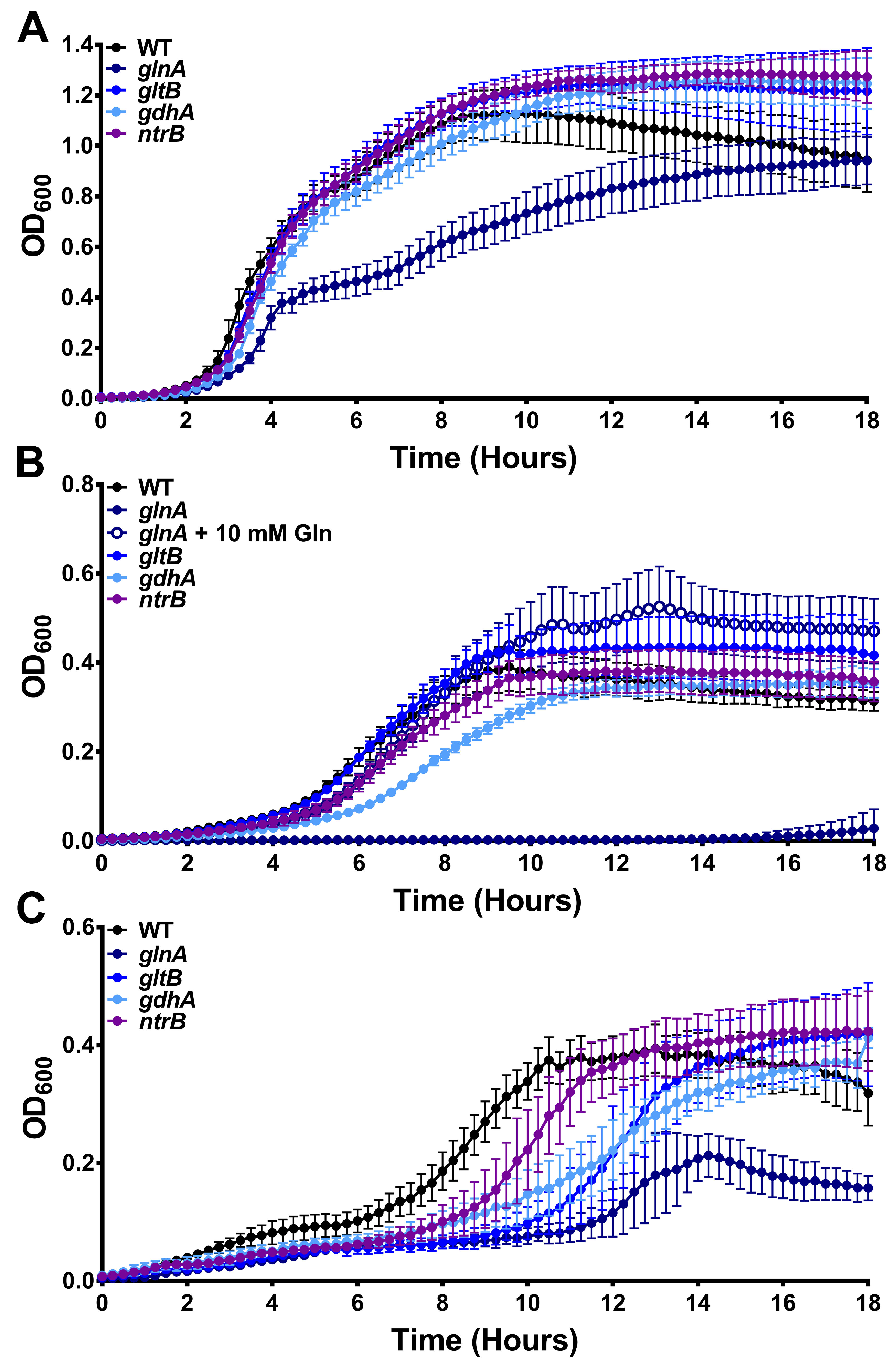

Supplement: S7 Fig — Growth of wild-type HI4320, glnA, gltB, ghdA, and ntrB strains was measured by optical density at 600 nm every 15 min over the course of 18 hours during incubation at 37°C with shaking in either LB (A), PMSM minimal medium (B) or RPMI (C). In addition, glnA was chemically complemented during growth in PMSM with 10 mM of L-glutamine. Error bars represent the mean ± standard deviation from two independent experiments, four replicates each. (TIF) [file ppat.1007653.s007.tif]

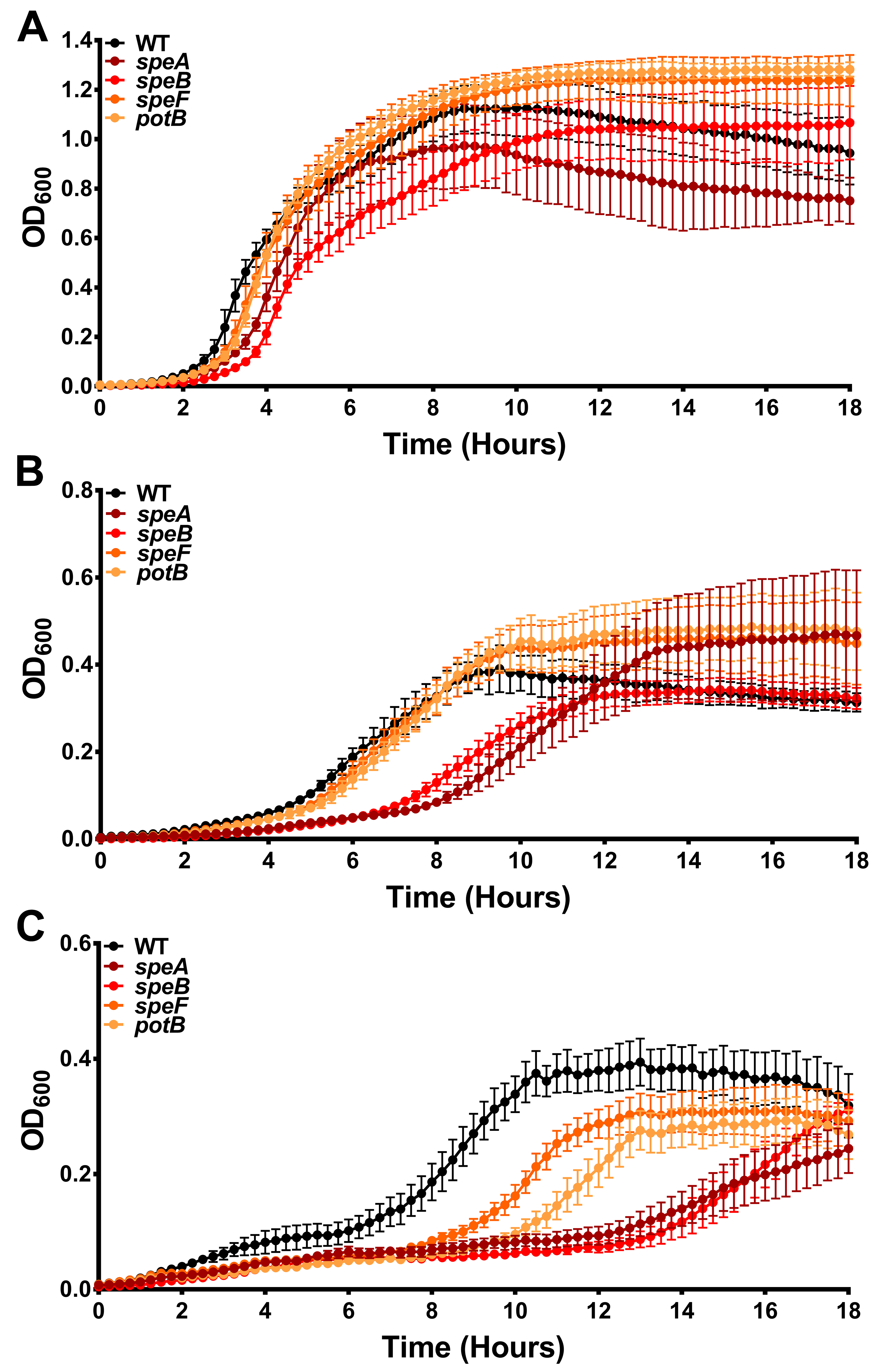

Supplement: S8 Fig — Growth of wild-type HI4320, speA, speB, speF, and potB strains was measured by optical density at 600 nm every 15 min over the course of 18 hours during incubation at 37°C with shaking in either LB (A), PMSM minimal medium (B) or RPMI (C). Error bars represent the mean ± standard deviation from two independent experiments, four replicates each. (TIF) [file ppat.1007653.s008.tif]
