# Supplementary material for: Twin arginine translocation, ammonia incorporation, and polyamine biosynthesis are crucial for Proteus mirabilis fitness during bloodstream infection
Source: PLoS Pathog. 2019 Apr 22;15(4):e1007653. doi: 10.1371/journal.ppat.1007653 (PMC6497324; doi:10.1371/journal.ppat.1007653)
Supplement: S8 Table — (DOCX) [file ppat.1007653.s016.docx]

| **Bacterial Strain** | **PMI Number** | **PMI_RS Number** | **Description** | **Reference** |
| --- | --- | --- | --- | --- |
| *Proteus mirabilis* HI4320 | NA | NA | WT CAUTI isolate | ([2](#_ENREF_2)) |
| *arnA* | PMI1045 | PMI_RS05080 | Bifunctional polymyxin resistance protein disrupted by Kan^R^ cassette | ([29](#_ENREF_29)) |
| *btuB* | PMI3246 | PMI_RS16050 | Vitamin B12 transporter disrupted by Kan^R^ cassette | This study |
| *cutC* | PMI2716 | PMI_RS13385 | Propanediol utilization protein disrupted by Kan^R^ cassette | This study |
| *asnA* | PMI3052 | PMI_RS15115 | Aspartate-ammonia ligase disrupted by Kan^R^ cassette | ([29](#_ENREF_29)) |
| *cvpA* | PMI1784 | PMI_RS08750 | Colicin V production protein disrupted by Kan^R^ cassette | ([29](#_ENREF_29)) |
| *hflK* | PMI3367 | PMI_RS16760 | Protease FtsH subunit disrupted by Kan^R^ cassette | ([29](#_ENREF_29)) |
| *tatA* | PMI3539 | PMI_RS17590 | Sec-independent protein translocase disrupted by Kan^R^ cassette | This study |
| *tatC* | PMI3541 | PMI_RS17600 | Sec-independent protein translocase disrupted by Kan^R^ cassette | This study |
| *glnA* | PMI2882 | PMI_RS14245 | L-glutamine synthetase disrupted by Kan^R^ cassette | ([46](#_ENREF_46)) |
| *gltB* | PMI3677 | PMI_RS18295 | Glutamate synthase large subunit disrupted by Kan^R^ cassette | This study |
| *gdhA* | PMI3008 | PMI_RS14875 | Glutamate dehydrogenase disrupted by Kan^R^ cassette | ([30](#_ENREF_30)) |
| *ntrB* | PMI2883 | PMI_RS14250 | Nitrogen regulation protein NR(II) disrupted by Kan^R^ cassette | This study |
| *speA* | PMI2094 | PMI_RS10315 | Arginine decarboxylase disrupted by Kan^R^ cassette | ([46](#_ENREF_46)) |
| *speB* | PMI2093 | PMI_RS10310 | Agmatinase disrupted by Kan^R^ cassette | ([46](#_ENREF_46)) |
| *speF* | PMI0307 | PMI_RS01480 | Ornithine decarboxylase disrupted by Kan^R^ cassette | ([46](#_ENREF_46)) |
| *potB* | PMI2736 | PMI_RS13485 | Spermidine/putrescine ABC transporter ATP-binding protein disrupted by Kan^R^ cassette | This study |
