# Supplementary material for: Twin arginine translocation, ammonia incorporation, and polyamine biosynthesis are crucial for Proteus mirabilis fitness during bloodstream infection
Source: PLoS Pathog. 2019 Apr 22;15(4):e1007653. doi: 10.1371/journal.ppat.1007653 (PMC6497324; doi:10.1371/journal.ppat.1007653)
Supplement: S9 Table — (DOCX) [file ppat.1007653.s017.docx]

| **Gene** | **Primer** | **Sequence** |
| --- | --- | --- |
| ***btuB*** | **IBS** | AAAAAAGCTTATAATTATCCTTAccttcccacaacGTGCGCCCAGATAGGGTG |
|  | **EBS1d** | CAGATTGTACAAATGTGGTGATAACAGATAAGTCcacaacatTAACTTACCTTTCTTTGT |
|  | **EBS2** | TGAACGCAAGTTTCTAATTTCGGTTgaaggTCGATAGAGGAAAGTGTCT |
|  | **Ver_F** | TGTAGCACAAAGTGGTGGTATAG |
|  | **Ver_R** | AATCGCATCGGAGCCATAAA |
| ***cutC*** | **IBS** | AAAAAAGCTTATAATTATCCTTACTCATCTTGAGCGTGCGCCCAGATAGGGTG |
|  | **EBS1d** | CAGATTGTACAAATGTGGTGATAACAGATAAGTCTTGAGCGATAACTTACCTTTCTTTGT |
|  | **EBS2** | TGAACGCAAGTTTCTAATTTCGGTTATGAGTCGATAGAGGAAAGTGTCT |
|  | **Ver_F** | GCTGGCAGAACGTTTAGTTTC |
|  | **Ver_R** | GAGTCACTACGCACTTCTTCTT |
| ***tatA*** | **IBS** | AAAAAAGCTTATAATTATCCTTAGCTTCCGTCAAAGTGCGCCCAGATAGGGTG |
|  | **EBS1d** | CAGATTGTACAAATGTGGTGATAACAGATAAGTCGTCAAAGGTAACTTACCTTTCTTTGT |
|  | **EBS2** | TGAACGCAAGTTTCTAATTTCGATTGAAGCTCGATAGAGGAAAGTGTCT |
|  | **Ver_F** | CATCGCTGTCATCGTAGTCTTAT |
|  | **Ver_R** | CTCTCAGTGGTTGACTTCTCTTC |
| ***tatC*** | **IBS** | AAAAAAGCTTATAATTATCCTTATCTGTCCCTGTGGTGCGCCCAGATAGGGTG |
|  | **EBS1d** | CAGATTGTACAAATGTGGTGATAACAGATAAGTCCCTGTGATTAACTTACCTTTCTTTGT |
|  | **EBS2** | TGAACGCAAGTTTCTAATTTCGGTTACAGATCGATAGAGGAAAGTGTCT |
|  | **Ver_F** | TCTCGGCACCTTTACTTGATAA |
|  | **Ver_R** | CACAGCAAGATGATCGCAATAG |
| ***gltB*** | **IBS** | AAAAAAGCTTATAATTATCCTTACCATTCGTGGATGTGCGCCCAGATAGGGTG |
|  | **EBS1d** | CAGATTGTACAAATGTGGTGATAACAGATAAGTCGTGGATTATAACTTACCTTTCTTTGT |
|  | **EBS2** | TGAACGCAAGTTTCTAATTTCGGTTAATGGTCGATAGAGGAAAGTGTC |
|  | **Ver_F** | CAGACTCAAGCTCACTGGATAA |
|  | **Ver_R** | CATAGCGACCGTCAGATAACA |
| ***ntrB*** | **IBS** | AAAAAAGCTTATAATTATCCTTAatttccgagcaaGTGCGCCCAGATAGGGTG |
|  | **EBS1d** | CAGATTGTACAAATGTGGTGATAACAGATAAGTCgagcaacaTAACTTACCTTTCTTTGT |
|  | **EBS2** | TGAACGCAAGTTTCTAATTTCGGTTgaaatcCGATAGAGGAAAGTGTCT |
|  | **Ver_F** | ACGGCGGATTTACCTGATAAC |
|  | **Ver_R** | TGTGCAACACGCTCTACAA |
| ***potB*** | **IBS** | AAAAAAGCTTATAATTATCCTTATATCCCCAATAGGTGCGCCCAGATAGGGTG |
|  | **EBS1d** | CAGATTGTACAAATGTGGTGATAACAGATAAGTCCAATAGAATAACTTACCTTTCTTTGT |
|  | **EBS2** | TGAACGCAAGTTTCTAATTTCGGTTGGATATCGATAGAGGAAAGTGTCT |
|  | **Ver_F** | CCGTGATGATGCGAATTTAGTC |
|  | **Ver_R** | CTGCTTCCAAACAGGGTTTATC |
