# Supplementary material for: Twin arginine translocation, ammonia incorporation, and polyamine biosynthesis are crucial for Proteus mirabilis fitness during bloodstream infection
Source: PLoS Pathog. 2019 Apr 22;15(4):e1007653. doi: 10.1371/journal.ppat.1007653 (PMC6497324; doi:10.1371/journal.ppat.1007653)
Supplement: S10 Table — (DOCX) [file ppat.1007653.s018.docx]

| SRA File | Barcode | Sample Name |
| --- | --- | --- |
| 91317 | ACGT | Input_1 |
| 91317 | AGTC | Input_2 |
| 91317 | ATCG | RPMI_1 |
| 91317 | CATG | RPMI_2 |
| 91317 | AGGA | Naïve_1 |
| 91317 | GTCA | Naïve_2 |
| 91317 | CGAT | APS_1 |
| 91317 | GTAC | APS_2 |
| 91329 | ACCA | Spleen 1 |
| 91329 | ACGT | Spleen 2 |
| 91329 | AGTC | Spleen 3 |
| 91329 | CATG | Spleen 4 |
| 91329 | CCAA | Spleen 5 |
| 91329 | CGAT | Spleen 6 |
| 91329 | GGGG | Spleen 7 |
| 91329 | GTAC | Spleen 8 |
| 91329 | TTAA | Spleen 9 |
| 91329 | AAAA | Spleen 10 |
| 91339 | ACCA | Liver 1 |
| 91339 | ACGT | Liver 2 |
| 91339 | AGTC | Liver 3 |
| 91339 | CATG | Liver 4 |
| 91339 | CCAA | Liver 5 |
| 91339 | CGAT | Liver 6 |
| 91339 | GGGG | Liver 7 |
| 91339 | GTAC | Liver 8 |
| 91339 | TTAA | Liver 9 |
| 91339 | AAAA | Liver 10 |

**Supplemental Table 10. Barcodes for de-multiplexing raw sequencing reads.**
